# The changing landscape of relapsed and/or refractory multiple myeloma (MM): fundamentals and controversies

**DOI:** 10.1186/s40364-021-00344-2

**Published:** 2022-01-09

**Authors:** José-Ángel Hernández-Rivas, Rafael Ríos-Tamayo, Cristina Encinas, Rafael Alonso, Juan-José Lahuerta

**Affiliations:** 1 Hospital Universitario Infanta Leonor, Departamento de Medicina, Universidad Complutense, Madrid, Spain; 2 Hospital Universitario Virgen de las Nieves, Instituto de Investigación Biosanitaria, Granada, Spain; 3 Hospital General Universitario Gregorio Marañón, Instituto de Investigación Sanitaria Gregorio Marañón, Madrid, Spain; 4 Hospital Universitario 12 de Octubre, Instituto de Investigación del Hospital Universitario 12 de Octubre, Madrid, Spain

**Keywords:** Multiple myeloma, Monoclonal antibodies, Proteasome inhibitors, Immunomodulatory drugs, New agents, Relapsed, Refractory

## Abstract

The increase in the number of therapeutic alternatives for both newly diagnosed and relapsed/refractory multiple myeloma (RRMM) patients has widened the clinical scenario, leading to a level of complexity that no algorithm has been able to cover up to date. At present, this complexity increases due to the wide variety of clinical situations found in MM patients before they reach the status of relapsed/refractory disease. These different backgrounds may include primary refractoriness, early relapse after completion of first-line therapy with latest-generation agents, or very late relapse after chemotherapy or autologous transplantation. It is also important to bear in mind that many patient profiles are not fully represented in the main randomized clinical trials (RCT), and this further complicates treatment decision-making. In RRMM patients, the choice of previously unused drugs and the number and duration of previous therapeutic regimens until progression has a greater impact on treatment efficacy than the adverse biological characteristics of MM itself. In addition to proteasome inhibitors, immunomodulatory drugs, anti-CD38 antibodies and corticosteroids, a new generation of drugs such as XPO inhibitors, BCL-2 inhibitors, new alkylators and, above all, immunotherapy based on conjugated anti-BCMA antibodies and CAR-T cells, have been developed to fight RRMM. This comprehensive review addresses the fundamentals and controversies regarding RRMM, and discusses the main aspects of management and treatment. The basis for the clinical management of RRMM (complexity of clinical scenarios, key factors to consider before choosing an appropriate treatment, or when to treat), the arsenal of new drugs with no cross resistance with previously administered standard first line regimens (main phase 3 clinical trials), the future outlook including the usefulness of abandoned resources, together with the controversies surrounding the clinical management of RRMM patients will be reviewed in detail.

## Background

Despite the increasing availability of more effective treatments for newly diagnosed multiple myeloma (MM) patients that provide longer disease-free periods, MM progresses in the vast majority of cases. Furthermore, the safety profile of modern drugs means that combinations of 3, 4 or more are now commonly used in the upfront treatment of MM in order to achieve a deeper, more prolonged response. For this reason, treating relapses after regimens that have included a proteasome inhibitor (PI), an immunomodulatory drug (IMID) and, sometimes, an anti-CD38 monoclonal antibody, can be challenging. The recent MM guidelines of the European Hematology Association-European Society of Medical Oncology (EHA-ESMO) include current second-line treatment options for patients who have received first-line drugs based on combinations of PI plus IMIDs or daratumumab-based therapies, as well as subsequent relapses [1]. This review provides updated information on novel agents for the treatment of relapsed/refractory MM patients and takes a critical look at the future outlook for clinicians working in this field.

## Basis for the clinical management of patients with relapsed/refractory multiple myeloma

The development of several generations of new drugs since the first decade of this century has radically improved the prognosis of MM [2]. These advances are the sum of the transfer of new drugs to the front-line setting and the impact of their use as rescue therapies. For patients with relapsed/refractory multiple myeloma (RRMM), this has meant an increase in the number of therapeutic options with no cross resistance with first-line or previous salvage therapies [3]. This scenario differs greatly from previous situations in which the lack of therapeutic options meant that the only alternatives were chemotherapy [4], second autologous stem cells transplantations (ASCT) [5], or even retreatment with bortezomib, thalidomide or lenalidomide previously used in the first line.

### The complexity of the clinical scenarios in RRMM

The large number of therapeutic alternatives currently available for MM patients gives rise to a wide range of possible clinical settings that cannot easily be covered by a single algorithm [6]. Currently, patients with MM achieve relapsed/refractory status (RR) after a range of therapeutic antecedents, ranging from primary refractoriness or early relapse after first-line treatment with latest-generation drugs, to very late relapse in patients treated with chemotherapy or autologous stem cell transplantation (ASCT) in the first line. Patients with RRMM previously treated with bortezomib (V) plus thalidomide (T) or lenalidomide (R) and dexamethasone (VTd or VRd) with ASCT who achieved median progression-free survival (PFS) of more than 5 years after front line treatments [7, 8] are not represented in the main RCT in RRMM, and are not considered in the algorithms proposed by most authors. This is also applicable to older RRMM patients, in whom first-line treatments with combination bortezomib, melphalan and prednisone, or the most recent monoclonal antibodies (mAbs), achieve a median PFS of more than 3 years [9]. Furthermore, in RRMM the variability of clinical scenarios increases as new lines of treatment are introduced after each episode of RRMM.

In general, the complexity of the contexts in which RRMM occurs is not adequately represented in the inclusion criteria of RCTs, in which the requirements regarding previous treatments are excessively generic, and frequently include euphemisms such as inclusion or exclusion due to prior “exposure” to a drug, without specifying dose, duration and response to treatment, associated drugs, or the interval from exposure to inclusion in the study, all of which are known to impact the efficacy of salvage treatments in MM. An additional confounding factor is the generalized inclusion requirement of “at least a progressive disease status with measurable disease" [10–19], which allows the inclusion of an undetermined percentage of patients in biological progression with a better prognosis than those showing clinical progression [20].

After all these considerations, the choice of treatment for a patient not represented in the main RCTs may be facilitated by resorting to some concepts that are well recognized in RRMM.

### Some keys to the choice of treatment

In MM, the adverse biological factors present at diagnosis (increased Beta2-microglobulin, hypoalbuminemia, anemia, kidney failure, high-risk genetic abnormalities, high lactate dehydrogenase, extension of active lesions on positron emission tomography/computed tomography (PET/CT), high proliferative index, etc.) are also important in RRMM [21]. However, their relevance in the choice of a rescue treatment is limited. Little information can be gained from RCTs, as patients presenting poor prognostic factors are usually excluded from these clinical studies. Interestingly, real-life data suggest the prognostic significance of performance status or cytogenetics in RRMM patients treated with novel therapies [22, 23].

In RRMM patients, the choice of salvage treatment involves not only an analysis of the response to and toxicity of previous treatments, but also a detailed evaluation of MM-related or unrelated comorbidities. Obviously, primary refractoriness to a drug rules out its use in the management of RRMM. Nevertheless, in MM the rechallenge of a drug previously used with success in the treatment of the disease is still possible, despite a certain loss of efficacy that increases in parallel with the number of previous treatment lines [24–26]. Factors that determine the efficacy of rescue therapies are, i) the use of drugs with proven efficacy in MM other than those previously used, ii) the number of accumulated RRMM episodes and iii) the length of the previous time to progression.

The current arsenal of drugs available for the treatment of RRMM improves the results of drug retreatment [27], which was hitherto the only therapeutic alternative [28, 29]. When choosing a therapeutic alternative it is important to take into consideration the results of RCTs and the possible cross-resistance between drugs from the same therapeutic group, such as the PIs bortezomib and carfilzomib [11], or the IMIDs lenalidomide and pomalidomide [30, 31].

The occurrence of RRMM episodes with increasingly reduced PFS periods will often exhaust the efficacy of new drug-based treatments. At this point, drug rechallenge [26], use of chemotherapy regimens [32, 33] or second ASCT [5] may halt progression for a limited period of time, though this may be long enough to allow patients to access to a new clinical study. Age, accumulated toxicities, and the anaplastic clonal evolution that is characteristic of very advanced stages of MM limit the efficacy of treatments in these final stages of the disease.

In a large study carried out by the Nordic Myeloma Study Group in patients treated in the 1990s [34], the duration of response to first-line treatment was identified as the prognostic factor with the greatest impact on RRMM. In this analysis, median overall survival (OS) in patients who relapsed after 6, 6 to 12, 12 to 24, and >24 months after ASCT was 3, 17, 28, and 37 months, respectively. Subsequent studies have confirmed that prolongation of the previous response or the length of the therapeutic pause before starting the next treatment favorably affects the prognosis of patients with RRMM after ASCT [35] or allogeneic transplant [36], non-transplanted patients treated with chemotherapy [37] or rescue-based therapies with new drugs [38, 39]. More recent studies confirm the adverse prognosis associated with early relapse [40]. Therefore, the previous time to progression or the duration and depth of the previous response, a prognostic factor systematically ignored in RCTs, is a determining factor in the choice of rescue treatments, and also in the increasingly frequent incidence of very late relapses as a result of the greater efficacy of first-line treatments [7, 8, 41]. RRMM patients previously treated with regimens that achieve long PFS periods could benefit from regimens used in the first-line setting and from new therapeutic opportunities. On the other hand, the time elapsed from the successful use of a drug in an advanced RRMM episode may favor rechallenge with drugs used previously, although obviously with limited results.

### When to treat

The established International Multiple Myeloma Working Group (IMWG) criteria for treatment indication in patients with RRMM requires meeting the Progressive Disease or Clinical Relapse criteria of the IMWG [42]. These criteria, maintained since 1998 without major changes, were established at a time when retreatment with chemotherapy or a second autologous transplant were the only available options for RRMM patients [33, 34, 36, 43]. In both cases, the chances of success were conditioned by the length of time before starting treatment, a subclinical waiting time that was considered beneficial for the patient's quality of life. At present, however, patients with RRMM can even be treated with several consecutive lines using drugs that show no cross-resistance with those used in prior lines. At the same time, follow-up techniques based on minimal residual disease (MRD), PET/CT or mass spectrometry have improved, allowing early and accurate prediction of therapeutic failure [44–46]. Importantly, the definition of failure should probably include not only the possibility of predicting progression or recurrence, but also insufficient tumor shrinkage marked by the stagnation of an insufficient response [47]. Although the formal exploration of this approach to RRMM is in its initial phases, data on the superiority of early treatment of RRMM compared to initiating therapy in clinical relapse have already been released, and the influence of tumor burden on treatment efficacy and tolerability in any of the evolutionary phases of MM is well known [48, 49].

## The arsenal of new drugs and therapies with no cross resistance with the therapeutic standards used in first line to date

PIs, IMiDs, MoAbs and corticosteroids constitute the cornerstone of treatment for patients with RRMM. Data from the main phase 3 RCT are summarized in Table 1.

**Table 1:** Summary of data from the main phase 3 studies with proteasome inhibitors, immunomodulatory drugs and monoclonal antibodies

Study	Arms	*N*	Doses and schedule	Follow-up (months)	Prior lines	PFS (months)	ORR (%)	≥CR (%)	OS (months)	>G3 AE (%)
ASPIRE [19]	KRd *vs.* Rd	792	K, 20/27mg/m^2^ BIW; R, 25 mg PO, days 1 and 21; 28-days cycle/s.	67.1	2 (1-3)	26.3 *vs.* 17.6 HR 0,690	87.1 *vs.* 66.7 OR 3.472	31.8 *vs.* 9.3	48.3 *vs.* 40.4 HR 0.794	87 *vs.* 83.3 AH, 6.4 *vs.* 2.3 CF, 4.3 *vs.* 2.1
ENDEAVOR [11]	Kd *vs.* Vd	929	K, 20/56 g/m^2 I^ IV, BIW, 28-days cycle; V, 1.3mg/m^2^ SC/IV, BIW 21-days cycle/s.	12	2 (1-3)	18.7 *vs.* 9.4 HR 0.533	76.9 *vs.* 62.6 OR 2.032	13 *vs.* 6	47.6 *vs.* 40 HR 0.791	An, 14 *vs.* 10 AH, 9 *vs.* 3 NP, 2 *vs.* 8
IKEMA [50]	IsaKd *vs.* Kd	302	Isa, 10mg/kg QW for 4w, then given Q2W; 28-days cycle/s.	20.7	2 (1-3)	NR *vs.* 19.15 HR 0.531	86.6 *vs.* 82.9 P 0.19	39.7 *vs.* 27.6	NA	76.8 *vs.* 67.2
CANDOR [51]	DKd *vs.* Kd	466	D, 8mg/kg IV, days 1-2 of cycle 1; 16 mg/kg QW, days 8, 15 and 22 first 2 cycles, then Q2W, cycles 3-6 and every Q4W thereafter; 28-days cycle/s.	17	2 (1-3)	NR *vs.* 15·8 HR 0.63	84 *vs.* 75 OR 1.9	29 *vs.* 10 (MRD, 18 *vs.* 4)	NA	82 *vs.* 74 TP, 24 *vs.* 16 AH, 18 *vs.* 13 Inf, 33 *vs.* 18 CF, 5 *vs.* 11
ARROW [16]	K QW *vs.* BIW	478	K, 20/70mg/m^2^ QW *vs.* 20/27mg/m^2^ BIW; 28-days cycle.	12.6 / 12	2 (46% *vs.* 64%) 3 (59% *vs.* 67%)	11.2 *vs.* 7.6 HR 0.69	62.9 *vs.* 40.8 OR 2.68	7 *vs.* 2	NM	68 *vs.* 62 CF, 3 *vs.* 4
ARROW2 (NCT03859427)	KRd QW *vs.* BIW	460	K, 20/ 56mg/m^2^ QW *vs.* 20/27mg/m^2^ BIW; 28-days cycle/s.	NM	1-3	NA^a^	NM	NM	NM	NM
MM 003 [52]	Pd *vs.* d	455	P, 4mg PO, days 1 and 21; 28-days cycle/s.	10	>2 (94.5% pts)	4 *vs.* 1.9 HR 0.48	31 *vs.* 10	1 *vs.* 0	12.7 *vs.* 8 HR 0.74	88.7 *vs.* 84.7
ICARIA-MM [10]	IsaPd *vs.* Pd	307	Pd ± Isa IV 10mg/kg, days 1, 8, 15, and 22 in cycle 1; days 1 and 15 in subsequent cycles; 28-days cycle/s.	11.6	≥2	11.53 *vs.* 6.47 HR 0.596	60.4 *vs.* 35.3	5 *vs.* 1^b^	NR	87 *vs.* 71 NP, 60.5 *vs.* 31.3
APOLLO EMN14 [53]	DPd *vs.* Pd	304	Pd ± D 16 mg/kg IV or 1,8 g SC QW (8 w), BIW (16 w), Q4W thereafter; 28-days cycle/s	16.9	>1	12.4 *vs*. 6.9 HR 0.63	69 *vs*. 46	25 *vs.* 4	NM	50 *vs.* 39 NP, 68 *vs.* 51 PN, 15 *vs.* 8
TOURMALINE-MM1 [15]	IRd *vs.* Rd	722	I, 4mg PO, days 1, 8 and 15, each 28-days cycle.	15	2 (1-3)	20.6 *vs.* 14.7 HR 0.74	78.3 *vs.* 71.5	12 *vs.* 7	NR	74 *vs.* 69
NCT03143049	PCd *vs.* Pd	120	Pd ± Cy 400mg days 1, 8, 15 and 22 each 28-days cycle.	NM	>1	NM	NM	NM	NM	NM
OPTIMISMM [31]	PVd *vs.* Vd	559	P, 4mg days 1-14 (each 21-days) +/- V, SC 1.3mg/m^2^ days 1, 4, 8, and 11 (cycles 1-8) and days 1 and 8 (>cycle 9); 21-days cycle/s.	15.9	>1	11.2 *vs.* 7.1 HR 0.61	82.2 *vs.* 50 OR 5.02	12.5 *vs.* 3.2	NR	57 *vs.* 42
CASTOR [54]	DVd *vs.* Vd	498	D, 16mg/kg IV QW, days 1, 8 and 15 of cycles 1-3; Q3W on day 1 of cycles 4-8, and Q4W thereafter	40	2 (1-10)	16.7 *vs.* 7.1 HR 0.31 (1 prior line, 27.0 *vs.* 7.9 HR 0.22)	85 *vs.* 63	30 *vs.* 10	NR	TP, 46 *vs.* 33 PN, 10 *vs.* 10 Inf, 29 *vs.* 19 Disc, 10 *vs.* 9
POLLUX [12]	DRd *vs.* Rd	569	D, 16mg/kg IV QW days 1, 8, 15 and 22 of cycles 1-2, 8 w and days 1 and 15 of cycles 3 – 6, 16 w and Q4W thereafter; 28-days cycle/s.	44	1 (1-11)	44.5 *vs.* 17.5 HR 0.44 (1 prior line, NR *vs.* 19.6 HR 0.42)	93 *vs.* 72	57 *vs.* 23 (MRD, 30.4 *vs.* 5.3)	NR	NP, 54.1 *vs.* 39.9 PN, 12 *vs.* 8.5 NP(F), 6.0 *vs.* 2.8
ELOQUENT-2 [14]	EloRd *vs.* Rd	646	Elo 10mg/kg IV, days 1, 8, 15 and 22 of cycles 1-2 and then on days 1 and 15 >cycle 3; 28-days cycle/s.	48	2 (1-4)	19.4 *vs.* 14.9 HR 0.71 (At 4 y, 21 *vs.* 14; H-risk, 15 *vs.* 7)	79 *vs.* 66	11 *vs.* 11	48 *vs.* 40	Inf, 33 *vs.* 26 LP, 79 *vs.* 49 NP, 36 *vs.* 45
ELOQUENT-3 [55]	EloPd *vs* Pd	117	Elo 10mg/kg days 1, 8, 15, and 22, cycles 1-2 and 20mg/kg on day 1 of each cycle thereafter; 28-days cycle/s.	9	3 (2-8)	10.3 *vs.* 4.7 HR 0.54	53 *vs.* 26	8 *vs.* 2	HR 0.62	NP, 13 *vs.* 27 Inf, 13 *vs.* 22
KEYNOTE-183 [56]	PembroPd *vs.* Pd	249	Pembro IV 200mg, Q3W; 28-days cycle/s.	8^c^	4 (2-4)	5.6 *vs.* 8.4	34 *vs.* 40	NM	At 6 m, 82 *vs.* 90	NP, 34 *vs.* 22 An, 17 *vs.* 13 PN, 14 *vs.* 13 AEDTH, 11 *vs.* 2

^a^Primary end point*;* ^b^ Underestimation due to interference with protein studies; ^c^ Premature study termination

*AE/AEDTH* adverse events/adverse events leading to death; *AH* arterial hypertension; *An* anemia: *BIW* twice a week; *CF* cardiac failure; *CR* complete response; *Cy* cyclophosphamide; *D* daratumumab; *d* dexamethasone; *Disc* discontinuation; *DKd* daratumumab/carfilzomib/dexamethasone; *DPd* daratumumab/pomalidomide/dexamethasone; *DRd* daratumumab/lenalidomide/dexamethasone; *DVd* daratumumab/bortezomib/dexamethasone; *Elo* elotuzumab*; EloPd* elotuzumab/pomalidomide/dexamethasone; *EloRd* elotuzumab/lenalidomide/dexamethasone; *G3* grade 3; *HR* hazard ratio; *H-risk* high risk; *I* ixazomib; *Inf* infection; *IRd* ixazomib/lenalidomide/dexamethasone; *Isa* isatuximab; *IsaKd* isatuximab/carfilzomib/dexamethasone*; IsaPd* isatuximab/pomalidomide/dexamethasone*; IV* intravenous; *K* carfilzomib; *Kd* carfilzomib/dexamethasone; *KRd* carfilzomib/lenalidomide/dexamethasone; *LP* lymphocytopenia; *MRD* minimal residual disease; *NM* not mature; *NP* neutropenia; *NP(F)* febrile neutropenia; *NR* not reached; *OR* odds ratio; *ORR* overall response rate; *OS* overall survival; *m* months; *P* pomalidomide; *PCd* pomalidomide/cyclophosphamide/dexamethasone; *Pd* pomalidomide/dexamethasone*; Pembro* pembrolizumab; *PembroPd* pembrolizumab/pomalidomide/dexamethasone; *PFS* progression-free survival; *PN* pneumonia; *PO* per orally; *Pts* patients; *PVd* pomalidomide/bortezomib/dexamethasone; *QW* once weekly; *Q2W* once every two weeks; *Q3W* once every three weeks; *Q4W* once every four weeks; *Rd* lenalidomide/dexamethasone*; TP* thrombocytopenia; *Vd* bortezomib/dexamethasone; *w* week; *y* years

### Proteasome inhibitors and new combinations

Carfilzomib (K) was approved in 2013 and, in combination with dexamethasone (Kd), has shown benefit compared to bortezomib plus dexamethasone (Vd) [11, 57]. When lenalidomide was added to Kd (KRd), overall response rate (ORR), PFS and OS improved compared to Rd, although the incidence of grade ≥ 3 adverse events (AEs) was similar [19, 58, 59]. Other combinations, such as the addition of pomalidomide to Kd (KPd), have been evaluated in 3 phase 1/2 RCTs (192 heavily treated patients who had a mean of 3.3 prior lines of therapy), reporting an ORR of 77.1% and a median PFS of 15.3 months [60–63]. Only one study reached an OS of 12 months [64]. So far, no phase 3 RCTs for this combination have been planned. Combinations of targeted therapy with CD38 MoAb, such as the combination of Kd plus daratumumab and Kd plus isatuximab, are a very interesting alternative IMiD-free approach, and have shown clinical benefits in terms of PFS with a favorable benefit-risk profile compared to Kd [50, 51].

The appropriate dosing schedule of K-containing combinations has also been explored in some studies. For example, administration of K once-weekly showed a favorable benefit-risk profile compared to the twice weekly schedule [16]. Other data also support the once-weekly dosing schedule, such as those obtained from triplet regimen with daratumumab in a phase 1 RCT (ORR = 84% and PFS not achieved with a short follow up of 4.5 months) [65] and with Rd [once-weekly (56 mg/m^2^) *versus* twice-weekly (27 mg/m^2^)], currently being tested in the ARROW2 trial (ClinicalTrials.gov number, NCT03859427).

Regarding other PIs, ixazomib added to Rd (Ixazomib-Rd) showed superior PFS compared to Rd [15, 66]. Furthermore, in a phase 2/3 trial, good tolerability and promising clinical activity has been observed when comparing this regimen with pomalidomide/dexamethasone (Pd) (ClinicalTrials.gov number, NCT03170882) [3].

### Pomalidomide and new combinations

Pomalidomide has shown favorable results in combination with other agents in clinical studies in which refractoriness and/or at least previous treatment with lenalidomide were the inclusion criteria. With dexamethasone (Pd), an improvement in OS was reported compared to dexamethasone alone [52], and the combination of pomalidomide with cyclophosphamide (PCd) has recently demonstrated better efficacy in phase 2 RCTs (median PFS 9.5 months) [64]. In combination with isatuximab, Pd showed clinical benefits compared to Pd alone in a phase 3 trial, with a higher incidence of some grade 3-4 AEs, but fewer AE-associated discontinuations [10, 67]. The combination with daratumumab (DPd) showed efficacy in a phase 1b study in 103 heavily treated patients (ORR of 60%, median PFS of 8.8 months and median OS of 17.5 months) [68] and the results of the phase 3 RCT that compared daratumumab plus Pd with Pd alone have been published recently [53]. The triplet PVd (pomalidomide, bortezomib and dexamethasone) was effective when compared to Vd in a population of patients of whom 100% had been exposed to lenalidomide and 71% were refractory to the treatment [31]. Finally, 2 ongoing phase 3 RCTs have been designed to evaluate the efficacy of the combination of Pd with nivolumab with or without elotuzumab (CheckMate-602; ClinicalTrials.gov number, NCT02726581) or with belantamab mafodotin (DREAMM8; ClinicalTrials.gov number, NCT04484623).

### Monoclonal antibodies and combinations

CD38 has so far been the most widely explored target in RRMM. Isatuximab (Isa) and daratumumab (D) are mAb’s against CD38 [69]. Isatuximab has a similar multimodal mechanism of action to daratumumab and elotuzumab, but binds a specific epitope on CD38; additionally, it is able to induce direct apoptosis without cross-linking and shows deeper inhibition of CD38 ectoenzymatic activity [70]. The phase 3 ICARIA-MM study observed that isatuximab combined with Pd in pomalidomide-naïve RRMM patients provided deeper and faster responses than Pd alone (ORR of 60.4% vs. 35.3%) with improved survival outcomes (median PFS 11.5 months), even for lenalidomide refractory patients [10]. Based on these results the European Commission approved the use of isatuximab in combination with pomalidomide and dexamethasone for the treatment of adult patients with relapsed/refractory multiple myeloma who have received ≥2 prior therapies, including lenalidomide and a proteasome inhibitor and have demonstrated disease progression on the last therapy [71]. Additionally, the combination of isatuximab with Kd in the IKEMA trial has recently showed promising results, with ORR 86.6%, including 29.6% of MRD-negative (10^-5^) patients, and median PFS not reached [hazard ratio (HR) 0.53, p = 0.007] after 20.7 months of follow-up [50]. In fact, isatuximab plus Kd has recently been approved by the European Medicines Agency for patients with RRMM and at least 1 prior therapy.

The combination of daratumumab with Vd [17, 72] or with Rd [12, 73, 74] have clearly demonstrated prolonged PFS, and DRd has achieved higher efficacy compared to Vd or Rd doublets in a network meta-analysis (with a reduction in the risk of death or progression of 81% and 63%, respectively) [75]. The combination of D with Kd is an effective IMiD-free option [51], and combined DPd treatment is currently being tested in the APOLLO EMN14 trial (ClinicalTrials.gov number, NCT03180736) [53, 63]. The incidence of grade >2 infusion-related reactions (IRRs) was 2%-3% and occurred almost exclusively after the first intravenous infusion. Subcutaneous administration has now received approval based on the results of a phase 3 RCT that showed a similar ORR than that obtained intravenously, but with fewer IRRs [76]. In combination with IMiDs, MOR-202, a novel anti-CD38 MoAb, has demonstrated an ORR ranging from 50%-65 % with lower IRR (7%) than daratumumab and isatuximab [77]. At present, a phase 3 trial comparing MOR-202 plus Rd *versus* Rd is ongoing.

Elotuzumab is a first-in-class IgG1-kappa that targets SLAMF7, and has already been approved in combination with Rd by the FDA [14, 55] and with Pd by the EMA [78]. The results of its combination with PIs are disappointing, although some RCTs testing elotuzumab plus KPd or elotuzumab plus PVd are currently ongoing. Finally, pembrolizumab, an anti-programmed death 1 (PD-1) checkpoint inhibitor, showed limited efficacy and higher mortality in the KEYNOTE-183 trial, leading to study termination [56].

### New drugs with other mechanisms of action

A summary of new drugs for RRMM management is included in Table 2. Melflufen, a melphalan prodrug with alkylating properties, induces rapid internalization of MM cells, and in combination with dexamethasone has recently shown promising results in phase 1/2 RCTs (O-12-M1 trial) [79]. A phase 3 study comparing its efficacy with Pd is currently ongoing. Increased ORR (≥80%) has been reported in combination with Vd or daratumumab (ANCHOR trial; ClinicalTrials.gov number, NCT03481556) [81].

**Table 2:** Main investigational drugs in RRMM

Trial/NTC Id	Arms / Phase	***N***	New agent	Number of prior lines	PFS (months)	ORR (%) CBR (%)	Median duration (months)	TTNT (months)	OS (months)	All /(>G3) AEs (%)
O-12-M1 [79]	Md Phase 1-2	81	Melflufen IV, 15, 25, 40 or 55mg (40mg: maximum tolerated dose)	4 (3-5)	5.7	31 49	8.4	NM	20.7	TP, 42 NP, 26 Infection, 8
NCT02899052	Venetoclax + Kd Phase 2	42	Venetoclax PO, 400 or 800mg	2 (1-3)		78 (100 % in t(11;14)				NP, 14 AH, 12
NCT03314181	Venetoclax + Dd Phase 1-2	24	Venetoclax, PO, various doses	3 (1-8)		92				NP, 13 AH, 8
BOSTON [80]	Vd ± selinexor Phase 3	404	Selinexor PO, 100mg oral, QW	1-3	13.93 vs. 9.46 HR 0.70	76 *vs*. 62	20.3 *vs*. 12.9	16.1 *vs*. 10.8	NR *vs*. 25	TP, 39 *vs*. 17 Fatigue, 13 *vs*. 1 Nausea, 8 *vs*. 0 PNP (≥ G2), 21 *vs*. 34
PANORAMA 1 [18]	Vd ± panobinostat Phase 3	768	Panobinostat PO, 20mg	1-3	11.99 vs. 8.08 HR 0.63	61 *vs*. 54	13.1 *vs*. 10.9		40.3 *vs*. 35.8	Diarrhea, 25 *vs.*8 Fatigue, 24 vs. 13 PN, 13 *vs*. 11 PNP, 18 *vs*. 15
NCT02384083	Filanesib + Pd, Phase 2	33	Filanesib IV, various doses	3 (2-6)	7	65	NM	NM	NM	NP, 60

*AEs* adverse events; *AH* arterial hypertension; *CBR* clinical benefit rate*; Dd* daratumumab/dexamethasone*; G2/G3* grade 2/grade 3; *HR* hazard ratio; *IV* intravenous; *Kd* carfilzomib/dexamethasone; *Md* melflufen/dexamethasone; *NM* not mature; *NP* neutropenia; *NR* not reached*; ORR* overall response rate; *OS* overall survival; *PFS* progression-free survival*; PN* pneumonia; *PNP* peripheral neuropathy; *Pd* pomalidomide/dexamethasone; *PO* per orally*; QW* once weekly*; TP* thrombocytopenia; *TTNT* time to next treatment; *Vd* bortezomib/dexamethasone

Venetoclax, a selective bcl2-inhibitor, has so far shown the best efficacy results in patients harboring t(11;14) [82]. Vd was tested in 291 patients in combination with venetoclax or placebo, showing improved PFS in the venetoclax arm: updated PFS results are 23.2 *vs*. 11.4 months, [HR 0.60 (0.43, 0.82)] and not reached (NR) *vs*. 9.3 months in patients with t(11;14) [HR 0.09 (0.02, 0.41)], but with a higher death rate [33.5 months *vs.* NR; HR 1.46 (0.91, 2.34)] mainly due to infection [83]. The study was terminated prematurely for this reason. A subgroup analysis showed a tendency toward better PFS and OS with venetoclax-Vd in t(11;14) and high BCL2 expression [83], a result that motivated the performance of the CANOVA study (ClinicalTrials.gov number, NCT03539744) that is currently testing this combination in this setting.

Selinexor, the first-in-class XPO1-inhibitor, has been approved in penta-refractory patients, showing improved efficacy in heavily treated patients (STORM study; ORR 26 %, PFS 3.7 months and OS 8.6 months) [65]. Furthermore, increased efficacy was reported when Vd was added (STOMP trial), [84] and recently the results of the phase 3 BOSTON trial (ClinicalTrials.gov number, NCT03110562) has confirmed this benefit in terms of PFS compared to Vd (HR 0.70, 95% CI 0.53–0.93; p=0·0075) [80]. Other regimens such as selinexor-Pd, selinexor-Kd or selinexor-daratumumab are being tested in phase 2 RCTs.

Panobinostat, a histone deacetylase (HDAC) inhibitor, received approval in 2015, and plus bortezomib plus dexamethasone showed benefit in PFS but no improvement in OS compared to Vd [85]. Moreover, toxicity was important, and dose reduction was needed in nearly 50% of patients.

Ibrutinib, a first-in-class covalent inhibitor of Bruton’s tyrosine kinase (BTK), does not have the same activity as in lymphoproliferative neoplasms, while the potent kinesin spindle protein (KSP) inhibitor, filanesib, has shown moderate efficacy when tested with PIs and IMiDs. Finally, JAK inhibitors (ruxolitinib) and cyclin dependent kinases (CDK) inhibitors (dinaciclib) are also being investigated in RRMM patients [86].

Cereblon E3 ligase modulators (CELMoDs) are a new class of agents that stimulate the immune system that have shown enhanced anti-MM activity in preclinical models, and are able to overcome lenalidomide/pomalidomide-resistance [87, 88]. In a phase 1b/2a trial with 66 highly pre-treated RRMM, iberdomide plus dexamethasone achieved ORR of 32.2% (35.3% for IMiD-refractory individuals), with a toxicity profile similar to that of IMiD (≥grade 3 AEs were mainly hematologic or infections) [89]. These results improved with the addition of bortezomib or daratumumab, achieving an ORR of 60.8% and 42.3%, respectively and maintaining a good safety profile [90]. The combination of CC-92480 plus dexamethasone was tested in a phase 1 trial in RRMM patients with high refractoriness rates [91], in which ORR was 54.5% at 1 mg QD 21/28 days, with 63% of responders being dual-IMiD refractory.

### Immunotherapy: Immunoconjugates, CAR-T cells and bispecific antibodies

Outcomes remain poor for triple-class–refractory patients (median OS ≤ 7-9 months), and there is no standard of care [92]. This has led to the need to develop new drugs with novel mechanisms of action to fill this gap.

B cell maturation antigen (BCMA) is an optimal target due to its restriction to B-cell lineage and overexpression in MM cells [93]. The antibody-drug conjugate belantamab mafodotin was the first anti-BCMA therapy approved. In a phase 2 trial in 97 patients, belantamab mafodotin at 2.5 mg/kg achieved an ORR of 32% (58% of responders showed ≥ very good partial response) with a median duration of response of 11 months [94]. Median PFS and OS were 2.8 months and 13 months, respectively, for a highly pre-treated population with a median of 7 prior lines of treatment and a marked refractoriness profile. The most common AE was keratopathy (70%), but this rarely led to discontinuation (1%). The most common grade ≥3 AEs were keratopathy (27%), anemia (20%) and thrombocytopenia (20%), with low incidence of grade 3 respiratory infections. IRRs were mostly grade 1-2 (no grade 4-5 and just one discontinuation). Studies evaluating synergies of belantamab mafodotin are ongoing [95, 96].

BCMA is now the most widely explored target for CAR-T cell therapies in MM, with more than 15 constructs being evaluated for RRMM patients (Table 3). Phase 2 trials with anti-BCMA CAR-T therapy [97, 109, 111, 112] confirm the promising results reported in earlier studies, with ORR ranging from 40% to 100% [98–108, 110, 113–116]. Additionally, some phase 1 studies have evaluated multi-antigen CAR-T strategies (targeting simultaneously BCMA and CD19 or CD38) [117, 118] as well as alternative targets (CD138, κLC, APRIL, GPRC5D, NY-ESO-1) with varying results [119–126].

**Table 3:** Summary of main trials evaluating anti-BCMA CAR-T cell therapy

Trials (***N***)	Construct	Baseline features	CAR-T cell dose	Lymphodepletion	RR	PFS (median)	CRS all/≥G3 (%)	ICANS all/≥G3 (%)
			**Phase 1/2**					
KarMMa/Ide-cel (***N*** = 128) [97]	Lentivirus Murine scFv 4-1BB/CD3ζ	6 y since diagnosis 6 prior lines HR cytogenetics: 35% Triple/Penta-refractory: 84/26%	150-450 x 10^6^ cells/kg	Cy/Flu	ORR 73%; ≥CR 33% MRD- at 10^-5^ 79% ^a^	8.8 m	84/5	18/3
EVOLVE/Orva-cel (***N*** = 62) [98]	Lentivirus Human scFv 4-1BB/CD3ζ, EGFRt	7 y since diagnosis 6 prior lines HR cytogenetics: 41% Triple/Penta-refractory: 94/48%	300-600 x 10^6^ cells	Cy/Flu	ORR 92%; ≥CR 36% MRD- at 10^-5^ 84%	NR	89/3	13/3
CARTITUDE-1/Cilta-cel (***N*** = 97) [99]	Lentivirus Llama VHH1-24-1BB/CD3ζ	5.9 y since diagnosis 6 prior lines HR cytogenetics: 23.7% Triple/Penta-refractory: 87.6/42.3% EMD: 13.4%	0.5-1.0 x 10^6^ cells/kg	Cy/Flu	ORR 96.9%; ≥CR 67% MRD- at 10^-5^ 54.6%	At 12-m, 76.6	94.8/5	16.5/2.1
PRIME/P-BCMA-101 (***N*** = 53) [100]	PiggyBac Human Centyrin 4-1BB/CD3ζ	4.9 y since diagnosis 8 prior lines Triple-refractory: 60%	51-1178 x 10^6^ cells Q2W cycles or combined with Rix or Lena	Cy/Flu	ORR 56.7% (out of 30 evaluable pts)	NR	17/0	3.8/3.8
LUMMICAR-2/CT053 **(*N*** **=** 20**)** [101]	Lentivirus Human scFv 4-1BB/CD3ζ	6 y since diagnosis 5 prior lines HR cytogenetics: 55% Triple/Penta-refractory: 85/50% EMD: 25%	1.5-3 x 10^8^ cells	Cy/Flu	ORR 94%; ≥CR 27.8% (out of 18 pts with ≥8 w of follow-up)	NR	78.9/0	15.8/5.3
			**Phase 1**					
NCI [102] **(*N*** **=** 16) [102]	γ-retrovirus Murine scFv CD28/CD3ζ	9.5 prior lines HR cytogenetics: 40%	9 x 10^6^ cells/kg	Cy/Flu	ORR 81%; ≥CR 12.5% MRD- at 10^-5^ 75%	EFS, 31 w	94/37.5	NR/19
CAR-T BCMA/UPenn **(*N*** **=** 25**)** [103]	Lentivirus Human scFv 4-1BB/CD3ζ	4.6 y since diagnosis 7 prior lines HR cytogenetics: 96% Penta-refractory: 44%	C1: 1-5 x 10^8^ cells C2: 1-5 x 10^7^ cells C3: 1-5 x 10^8^ cells	C1: No C2: Cy C3: Cy	C1: ORR 44%; ≥CR 9% C2: ORR 20%; ≥CR 0% C3: ORR 64%; ≥CR11%	C1: 65 d C2: 57 d C3: 125 d	C1: 89/33 C2: 60/0 C3: 100/45	C1: 33/22 C2: 20/0 C3: 36/9
CRB-402/bb21217 **(*N*** **=** 69**)** [104]	Lentivirus Murine scFv 4-1BB/CD3ζ bb007 PI3K inh	5.7 y since diagnosis 6 prior lines HR cytogenetics: 33% Triple-refractory: 64%	C1: 150 x 10^6^ cells C2: 300 x 10^6^ cells C3: 450 x 10^6^ cells Exp: 450 x 10^6^ cells	Cy/Flu	C1: ORR 83%; ≥CR 42% C2: ORR 43%; ≥CR 14% C3: ORR 57%; ≥CR 29% Exp: ORR 84%; ≥CR 32%	NR	70/4	16/4
MSKCC/MCARH171 **(*N*** **=** 11**)** [98]	γ-retrovirus Human scFv 4-1BB/CD3ζ, EGFRt	6 prior lines HR cytogenetics: 82%	72-818 x 10^6^ cells	Cy or Cy/Flu	ORR 64% (≥CR 0%)	NR	60/20	10/0
FHCRC/FCARH143 **(*N*** **=** 7**)** [105]	Lentivirus Human scFv 4-1BB/CD3ζ, EGFRt	8 prior lines HR cytogenetics: 100%	50-800 x 10^6^ cells	Cy/Flu	ORR 100% (≥CR 36%)	NR	91/0	9/0
HRAIN Biotechnology **(*N*** **=** 20**)** [106]	γ-retrovirus Murine scFv 4-1BB/CD3ζ, EGFRt	5.5 prior lines	9 x 10^6^ cells/kg	Cy/Flu	ORR 79% (≥CR 45%)	15 m	45/5	NR/7
FHVH-BCMA **(*N*** **=** 12**)** [107]	γ-retrovirus FHVH33 4-1BB/CD3ζ, EGFRt	6 prior lines HR cytogenetics: 58%	0.75-3 x 10^6^ cells/kg	Cy/Flu	ORR 83% (≥CR 17%)	NR	91.7/8.3	25/8.3
CT103A **(*N*** **=** 14**)** [108]	Lentivirus Human scFv 4-1BB/CD3ζ	4 prior lines	1-6 x 10^6^ cells/kg	Cy/Flu	ORR 100% (≥CR 71%)	NR	94.4/28	0/0
C-CAR088 **(*N*** **=** 23**)** [109]	Lentivirus Human scFv 4-1BB/CD3ζ	4 prior lines	1-6 x 10^6^ cells/kg	Cy/Flu	ORR 95.7% (≥CR 43.5%)	At 6-m: 65.1%	91.3/4.3	4.3/0
UNIVERSAL/ ALLO-715 **(*N*** **=** 31**)** [110]	Lentivirus Human scFv Rix-RD 4-1BB/CD3ζ Allogeneic TC with disrupted TCRα and CD52	5.4 y since diagnosis 5 prior lines HR cytogenetics: 48%	40-480 x 10^6^ cells	ALLO-647/ Cy +/- Flu	ORR 62% (≥VGPR 38%) at 320 x10^6^ cells (*n*=13)	NR	45/0	0/0

^a^MRD is referred to patients with ≥CR.

*C* cohort; *CR* complete response; *CRS* cytokine-release syndrome; *Cy* cyclophosphamide; *d* days; *EFS* event-free survival; *EMD* extramedullary disease; *Exp* expansion; *FHVH* fully human heavy-chain variable domain; *Flu* fludarabine; *G* grade; *HR* high-risk; *ICANS* immune-effector cell-associated neurotoxicity syndrome; *Lena* lenalidomide; *m* months; *MRD* minimal residual disease; *NR* not reported; *ORR* overall response rate; *PFS* progression-free survival; *Rix* rituximab; *Rix-RD* rituximab recognition domain; *RR* response rate; *scFv* single chain variable fragment, *TC* T-cells; *TCR* T cell receptor; *VGPR* very good partial response; *VHH* variable domain of heavy chain; *w* weeks; *y* year

Last but not least, bispecific antibodies in RRMM can be incorporated into the therapeutic arsenal of drugs against RRMM. Recently, results of bispecific CD3/BCMA antibodies as teclistamab and elranatamab, and bispecific CD3/BFCR4350A (talquetamab) and CD3/FcRH5 (cevostamab) antibodies have shown a manageable safety profile and encouraging results [21, 127–129].

## Controversies surrounding the clinical management of patients with RRMM

The clinical value of earlier treatments for RRMM: conventional and high-dose chemotherapy with autologous transplantation, lenalidomide and bortezomib

Conventional chemotherapy (CC) remains a cornerstone in the current therapeutic approach, particularly with new agents. However, CC is gradually being side-lined due to the better risk/benefit ratio of the new combinations [130]. Bridging therapy (when cytoreduction is urgently needed before more definitive therapy is planned) is one of the most common indications for CC, especially in certain circumstances, such as aggressive extramedullary disease or secondary plasma cell leukemia [131].

The role of high-dose chemotherapy and salvage ASCT (sASCT) is currently questioned in today’s RRMM treatment scenario, and must be placed in the context of the re-induction used [132]. The feasibility and efficacy of sASCT is mainly derived from retrospective studies [133–146] (Table 4). So far, two phase 3 trials have demonstrated the benefit of sASCT: the Myeloma X [5, 148] and the ReLApsE studies [147], with median PFS of 19 and 20.7 months, respectively.

**Table 4:** Recent studies in salvage autologous stem cell transplantation (sASCT**)** in relapsed/refractory multiple myeloma

Study	Type of study	*N*	ORR (%)	mPFS (months)	mOS (months)
Dhakal B et al. 2020 [135]	Retrospective (no tandem)	975	-	12	NR 1-y OS 94%
Goldschmidt H et al. 2020 [147]	Phase 3 (ReLApsE)	139 (sASCT) *vs.* 138 (Rd c.)	82/71	20.7/18.8 (ITT)	NR/62.7 (ITT)
Manjappa S et al. 2018 [140]	Retrospective (no tandem)	63 (30m, 33 no m)	92	13.8/20.3	-
Gössi U et al. 2018 [138]	Retrospective	86 (sASCT *vs.* CTna)	70	30.2/13	129.6/33.5
Veltri LW et al. 2017 [145]	Retrospective	233 (105 DR)	81	17.6	48
Nieto Y et al. 2017 [134]	Phase 2	74/184 (GBMF *vs.* MF)	-/70	15.1/9.3	37.5/23
Zannetti BA et al. 2017 [146]	Retrospective	66	94	17	43
Singh Abbi KK et al. 2015 [144]	Retrospective	75	82	10.1	22.7
Cook G et al. 2016 [5]	Phase 3 (Myeloma X)	89/85 (sASCT *vs.* CFx12w)	83/75	19/11	67/52
Sellner L et al. 2013 [142]	Retrospective	200	80.4	15.2	42.3
Michaelis LC et al. 2013 [141]	Retrospective (no tandem)	187	68	11.2	30
Gonsalves WI et al. 2013 [137]	Retrospective	98	86	10.3	33
Auner HW et al. 2013 [133]	Retrospective	83	-	15.5	31.5
Lemieux E et al. 2013 [139]	Retrospective	81	93	18	48
Shah N et al. 2012 [143]	Retrospective	44	90	12.3	31.7
Gertz MA et al. 2000 [136]	Retrospective	64 (14 PR, 20 RR, 30 Re)	34 (CR)	11.4	19.6

*CTna* conventional therapy including novel agents; *DR* double refractory (IPs & IMiDs); *GBMF* gemcitabine busulfan and melphalan; *ITT* intention-to-treat- population; *M* previous maintenance; *MF* melphalan; *mOS* median overall survival; *mPFS* median progression-free survival; *NR* not reached; *PR* primary refractory; *Rd* lenalidomide and dexamethasone, continuous; *Re* relapse off therapy; *RR* refractory relapse; *sASCT* salvage autologous stem cell transplantation; *w* weeks

### Selecting the best treatment for MM patients previously treated with lenalidomide in their first recurrence/progression

Most patients will have received lenalidomide when relapse occurs as a result of front-line therapy. In this context, at least 3 scenarios are possible: patients treated with upfront ASCT and lenalidomide as maintenance therapy [149]; patients not eligible for ASCT and treated with lenalidomide-containing regimens [41, 150–152] (lenalidomide administered until progression); and a third scenario with a small number of patients treated in first-line with lenalidomide and relapsing after a period without treatment. According to IMWG criteria, patients are defined as refractory to lenalidomide when presenting a non-responsive disease while on a lenalidomide-containing therapy or have progressed within 60 days of the last date of lenalidomide uptake [153]. However, from a clinical point of view there is no consensus on the management of patients with refractoriness to lenalidomide. Most experts recommend lenalidomide-free therapy for clinical progressions, regardless of the duration of response or the dose of lenalidomide [154]. In the case of a non-aggressive relapse in patients on low-dose lenalidomide, it is unclear whether response can be achieved with full-dose lenalidomide added to dexamethasone, or even by adding a third drug [154]. This is compounded by the fact that this population has been excluded from phase 3 RCTs evaluating lenalidomide-combinations, so the use of a lenalidomide-free triplet is also a better option in these patients. There is also evidence that a prolonged response to lenalidomide is associated with a better response to subsequent treatments after resistance to lenalidomide [39].

The lenalidomide-free options available so far were Kd and daratumumab plus VD (DVd) [11, 72]; however, these were less effective in patients previously treated with lenalidomide, and their efficacy following first relapse remains unknown due to the small number of patients included in clinical studies [11, 72, 155]. More effective carfilzomib- and pomalidomide- containing regimens are being incorporated in this setting. These have been assessed in studies in which a more representative population of patients already exposed to lenalidomide were included, especially in regimens containing pomalidomide, in which previous exposure to lenalidomide and even refractoriness was mandatory for recruitment (Table 5). Therapy with anti-CD38 plus Kd or Pd should be considered [159]. Daratumumab plus Kd (DKd) [51, 160], isatuximab plus Kd (Isa-Kd) [50], Isa-Pd [10] and DPd [53] have recently shown efficacy in exposed and refractory patients to lenalidomide in phase 3 RCTs. On first relapse after lenalidomide, there are only available data with DPd in a phase 2 study [156] and with anti-CD38-free therapy; in this context, pomalidomide plus PI (KPd) [157] or Vd (PVd) [31, 158] have shown efficacy. In conclusion, based on the current evidence, pomalidomide could be the key salvage combination in patients refractory to lenalidomide, and adding antiCD38 or carfilzomib will probably improve efficacy.

**Table 5:** Summary of patients enrolled in phase 2/3 clinical trials according to previous exposure to bortezomib or lenalidomide or refractoriness to lenalidomide

Study	Regimen	Phase	V-ex (%)	V-ref (%)	R-ex (%)	R-ref (%)	R-ref in 1L ***n*** (%)	ORR/CR (%)	ORR/CR R-ref (%)	PFS (months)	PFS R-ex (months)	PFS R- ref (months)	PFS R-ref in 1L (months)
ENDEAVOR [11]	Kd	3	54	4	38	25	32 ^a^	77/13	---	18.7	12.9	8.6	15.6 ^a^
CASTOR [72]	DVd	3	65	0	36	24	----	85/30	---	16.7	9.5	7.8 *vs.* 4.9 HR 0.44	----
OPTIMISMM [31]	PVd	3	72	9	100	71	129 (23) ^b^	82.2/15.7	----	11.2	11.2	9.5	17.8 ^b^
CANDOR [51]	DKd	3	92	29	42	33	----	84/31.8	----	NR *vs.* 15.8 HR 0.63	NR *vs.* 12.1 HR 0.52	NR *vs.* 11.1 HR 0.45	----
MM-014 [156]	DPd	2	78	--	100	75	70 (62.5)	----	----	NR	NR	21.8	NR
APOLLO EMN14 [53] ^c^	DPd	3	100	47	100	79	16 (11)	69/25		12.4 *vs.* 6.9 HR 0.63	NR	9.9 vs. 6.5 HR 0.66	---
IKEMA [50]	IsaKd	3	93	31	----	32	----	86.6/39.7	----	NR HR 0.53	----	NR HR 0.6	----
EMN011 [157] ^d^	KPd	2	100	100	100	100	100	87/65	87/65	18	18	18	18
ICARIA-MM [10]	IsaPd	3	100	77	100	94	----	60.4	----	11.53 HR 0.59	----	HR 0.5	----

^a^Data from ENDEAVOR and Champion-1 [155]; ^b^ Dimopoulos et al. (2018) [158]; ^c^ Only 11% after 1L in APOLLO; ^d^ Prior first line treatment in EMN02/H095 trial;

*CR* complete response; *DKd* daratumumab/carfilzomib/dexamethasone*; DPd* daratumumab/pomalidomide/dexamethasone*; DVd* daratumumab/bortezomib/dexamethasone*; ex* exposed; *HR* hazard ratio; *IsaPd* isatuximab /pomalidomide/dexamethasone; *Kd* carfilzomib/dexamethasone; *KPd* carfilzomib/pomalidomide/dexamethasone; *1L*, first line; *NR* not reached; *ORR* overall response rate; *PFS* progression-free survival; *R* lenalidomide; *ref* refractory; *V* bortezomib; *y* years

Finally, in the third scenario (patients who relapse after a long lenalidomide-free period), the treatment with lenalidomide triplets [DRd, KRd, VRd, Isa-Rd or elotuzumab-Rd] could be an option. Although no head-to-head comparison studies are available, DRd is probably the best treatment option in terms of PFS [3]; however, as mentioned above, the number of patients already exposed to lenalidomide included in these RCTs was small, so lenalidomide triplets were optimal when the only alternative was DVd or Kd. Nevertheless, in the context of the next available alternative, even in the third scenario, a lenalidomide-free combination could be a better choice.

### First-line of rescue vs. more advanced phases and double refractory patients in the main phase 3 trials for RRMM patients

A gradual decrease has been observed in the number of patients with improved clinical outcomes after each subsequent line of therapy, and the likelihood of obtaining a deep response is progressively slimmer. Therefore, an in-depth analysis of the results of the aforementioned main phase 3 trials in specific subpopulations according to prior lines of therapy will show what to expect from each drug-combination and how to maximize their performance. Overall, as expected, most treatments performed better at first relapse. Since slightly different inclusion criteria are used in these studies, direct comparisons are not always possible (Table 6). Furthermore, since subanalysis based on the number of prior lines were not performed in many studies, no information on the major prognostic factors that characterize each cohort is available. A cursory analysis would lead to a hypothetically longer PFS in patients with 2-3 prior lines of treatment that were rescued with Rd compared to those treated with Pd. Nevertheless, the latter was considered in early relapse after treatment with lenalidomide and bortezomib. Another important aspect is the difference in dosing schedules for the same control arms across different clinical trials. Thus, treatment with Vd was limited to 8 cycles in CASTOR [17, 54] and 12 cycles in PANORAMA-1 [18] and maintained until progression or intolerance in ENDEAVOR [11, 161, 162] and OPTIMISMM, [31] which limits comparisons based on HR. Similarly, Vd-based therapies provide shorter PFS than Rd-based regimens after first and second/third relapses, even though the latter was maintained as part of triplet therapy indefinitely [55, 58, 66, 72]. Treatment was discontinued at some point in both the CASTOR and PANORAMA-1 trials [17, 18, 54]. In contrast, PVd was maintained in the OPTIMISMM trial, with PFS outcomes at first relapse in the range of Rd-based therapies [31]. New triplets including MoAbs have not achieved median PFS, but considering their long follow-up (≥17 months), outcomes are expected to be promising [50, 51, 156].

**Table 6:** Progression-free survival outcomes in the main advanced-phase clinical trials for RRMM patients according to number of prior lines of therapy

	Regimen – Trial	Median PFS, months			Key Inclusion Criteria
		1 prior line	2-3 prior lines	>3 prior lines	
**Vd**	Vd – CASTOR [54, 72]	**7.9** (*n* = 113)	**6.3** (*n* =106)	**5.4** (*n* = 28)	-PR to ≥1 prior line -No refractoriness to PI -No prior anti-CD38
	Vd - PANORAMA-1 [18, 85]	**8.5** (*n* = 174)	**7.6** (*n* =207)	-	-No refractoriness to PI -No prior HDAC inhibitor
	Vd – OPTIMISMM [31]	**11.63** (*n* = 115)	7.10* (*n* = 163)	-	-No refractoriness to V at 1.3 mg/m2 BIW -Prior R, no prior P
	Vd – ENDEAVOR [161, 162]	**10.1** (*n* = 232)	**8.4** (*n* = 233)	-	-PR to ≥1 prior line -Prior PI allowed if ≥PR and ≥6 m since last dose
**Rd**	Rd – ASPIRE [58]	**17.6** (*n* = 157)	**16.7** (*n* = 239)	-	-PR to ≥1 prior line -No refractoriness to V, no prior K -No prior PD during 3 first m of Rd or any PD if Rd was the last therapy
	Rd – POLLUX [73, 74]	**19.6** (*n* = 146)	**15.7** (*n* = 118)	**17.1** (*n* = 19)	-PR to ≥1 prior line **-**No prior anti-CD38 -No refractoriness to R
	Rd - TOURMALINE-1 [66]	**16.6** (*n* = 213)	**12.9** (*n* = 149)	-	-No refractoriness to R or PI (refractoriness to thali is allowed)
	Rd - ELOQUENT-2 [55] ^a^	**12.1** (*n* = 97)	**13.1** (*n* = 65)	-	-Prior R is allowed if no refractoriness, ≥PR, no more than 9 prior cycles and at least 9 m before progression
	Rd – ELOQUENT-2 [55] ^b^	**19.4** (*n* = 62)	**14.9** (*n* = 101)	-	-Prior R is allowed if no refractoriness, ≥PR, no more than 9 prior cycles and at least 9 m before progression
**Pd**	Pd – MM-010 [13]	-	**3.9** (*n* = NR)	**4.6** (*n* = NR)	-≥2 prior lines including V and R -At least 4 cycles of alkylator or PD after at least 2 cycles or ASCT -PD within 6 m of discontinuation after PR with V and R -No prior P
	Pd – ICARIA-MM [10, 163]	-	**7.8** (*n* = 101)	**4.3** (*n* = 52)	-At least prior ≥MR -At least 2 prior lines including 2 cycles of a PI and R and PD within 6 m of discontinuation after PR -No prior P, no refractoriness to anti-CD38
	Pd – APOLLO EMN14 [53]	**12.6** (*n* = 18)	**6.5** (*n* = 113)	**6.6** (*n* = 22)	-At least 1 prior line including lena and a PI -PR to ≥1 prior line -No prior P, no prior anti-CD38
	Pd – ELOQUENT-3 [78]	-	**4.8** (*n* = 36)	**4.3** (*n* = 21)	-At least 2 prior lines including 2 cycles of a PI and R and PD within 6 m of discontinuation after PR -Refractory to PI and R -No prior P
**Kd**	Kd – ENDEAVOR [161, 162]	**22.2** (*n* = 232)	**14.9** (*n* = 232)	-	-PR to ≥1 prior line -Prior PI allowed if ≥PR and ≥6 m since last dose
	Kd – CANDOR [51, 164]	**21.3** (*n* = 67)	**12.5** (*n* =87)	-	-PR to ≥1 prior line -Prior K and/or anti-CD38 allowed if ≥PR, no refractoriness and >6 m since last dose
	Kd – IKEMA [50]	**NA** (*n* = 55)	**16.2** (*n* = 68)	-	-No prior K -No refractoriness to prior anti-CD38
**Vd based**	DVd – CASTOR [54, 72]	**27** (*n* = 122)	**9.8** (*n* = 107)	**8.1** (*n* = 22)	-PR to ≥1 prior line -No refractoriness to PI -No prior anti-CD38
	PanoVd – PANORAMA-1 [18, 85]	**12.3** (*n* = 178)	**12** (*n* = 209)	-	-No refractoriness to PI -No prior HDAC inhibitor
	PVd – OPTIMISMM [31]	**20.73** (*n* = 111)	11.2* (*n* = 170)	-	-No refractoriness to V at 1.3 mg/m2 BIW -Prior lena, no prior poma
	VeneVd – BELLINI [83]	**22.4** (*n* = 135)	**NA** (*n* = 156)	-	-No refractoriness or intolerance to prior PI -At least PR to any prior PI -At least 60-days PI-treatment-free interval
	XVd – BOSTON [80]	**16.6** (*n* = 99)	**11.8** (*n* = 96)		-No refractoriness or intolerance to prior PI -At least PR to any prior PI -At least a 6-month PI-treatment-free interval
**Rd based**	KRd – ASPIRE [58]	**29.6** (*n* =1 84)	**25.8** (*n* = 212)	-	-PR to ≥1 prior line -No refractoriness to V, no prior K -No prior PD during 3 first m of Rd or any PD if Rd was the last therapy
	DRd – POLLUX [73, 74]	**53.3** (*n* = 149)	**28.9** (*n* = 123)	**38.8** (*n* = 14)	-PR to ≥1 prior line **-**No prior anti-CD38 -No refractoriness to R
	IRd - TOURMALINE-1 [66]	**20.6** (*n* = 212)	**NA** (FU 14.8 m) (*n* = 148)	-	-No refractoriness to R or PI (refractoriness to thali is allowed)
	EloRd - ELOQUENT-2 [55] ^a^	**15.8** (*n* = 103)	**13.1** (*n* = 58)	-	-Prior R is allowed if no refractoriness, ≥PR, no more than 9 prior cycles and at least 9 m before progression
	EloRd - ELOQUENT-2 [55] ^b^	**30.6** (*n* = 48)	**25** (*n* = 112)	-	-Prior R is allowed if no refractoriness, ≥PR, no more than 9 prior cycles and at least 9 m before progression
**Pd based**	IsaPd - ICARIA-MM [10, 163]	-	**12.3** (*n* = 102)	**9.4** (*n* = 52)	-At least prior ≥MR -At least 2 prior lines including 2 cycles of a PI and R and PD within 6 m of discontinuation after PR -No prior P, no refractoriness to anti-CD38
	DPd – MM-014 [156]	**1y-PFS 78.8%** (FU 17.2 m) (*n* = 70)	**1y-PFS 69.0%** (FU 17.2 m) (*n* = 42) ^c^	-	-1-2 prior lines with at least 2 cycles of R -No prior P, no prior D
	DPd – APOLLO [53]	**14.1** (*n* =16)	**10.7** (*n* =114)	**19.3** (*n* =21)	- No prior P, no prior anti-CD38
	EloPd - ELOQUENT-3 [78]	-	**10.3** (*n* = 36)	**10.3** (*n* = 24)	-At least 2 prior lines including 2 cycles of a PI and R and PD within 6 m of discontinuation after PR -Refractory to PI and R -No prior P
**Kd based**	DKd – CANDOR [51, 164]	**NA** (FU 17.2 m) (*n* = 133)	**24.2** (*n* = 179)	-	-PR to ≥1 prior line -Prior K and/or anti-CD38 allowed if ≥PR, no refractoriness and >6 m since last dose
	IsaKd – IKEMA [50]	**NA** (FU 20.7 m) (*n* = 80)	**NA** (FU 20.7 m) (*n* = 99)	-	-No prior K -No refractoriness to prior anti-CD38

*Result for ITT population; ^a^ <3.5 years from diagnosis; ^b^ ≥3.5 years from diagnosis; ^c^ Just 2 prior lines of therapy

*ASCT* autologous stem cell transplantation; *BIW* twice in a week; *D* daratumumab; *EloPd* elotozumab/pomalidomide/dexamethasone; *EloRd* elotozumab/pomalidomide/dexamethasone *FU* follow-up; *HDAC* histone deacetylase; *IRd* ixazomib/lenalidomide/dexamethasone; *Isa* isatuximab; *IV* intravenous; *K* carfilzomib; *Kd* carfilzomib/dexamethasone; *m* months; *MR* minimal response; *NA* not achieved; *NR* not reported; *P* pomalidomide; *PanoVd* panobinostat/bortezomib/dexamethasone; *PD* progressive disease; *Pd* pomalidomide/dexamethasone; *PFS* progression-free survival; *PI* proteasome inhibitor; *PR* partial remission; *R* lenalidomide; *Rd* lenalidomide/dexamethasone; *Thali* thalidomide; *V* bortezomib; *Vd* bortezomib/dexamethasone; *VeneVd* venetoclax/bortezomib/dexamethasone; *XVd* selinexor/bortezomib/dexamethasone

Patients refractory to PIs and IMiDs have shown limited survival (median OS of 5-10 months), but no recent data on the benefit obtained with new therapies are available [165, 166], and there is no clear consensus on the meaning of double refractoriness. While double refractoriness was originally defined as refractoriness to bortezomib and lenalidomide, alternative drugs (carfilzomib, pomalidomide) are sometimes used in the first line setting. Thus, several studies define double-refractory patients as those who are refractory to any PI plus lenalidomide or to any PI plus any IMiD. Nevertheless, these double-refractory patients are not the same, and their outcomes could be different. Additionally, the front-line use of anti-CD38 MoAbs is becoming more frequent, and it is not uncommon to find patients who are refractory to anti-CD38 following an earlier relapse. Thus, the traditional definition of double-refractoriness is fast becoming triple-refractoriness. This scenario is not included in many studies, particularly earlier ones, and the heterogeneity makes it difficult to obtain precise information about outcomes in double-refractory patients.

Preliminary results with pomalidomide and daratumumab in patients relapsing after bortezomib and lenalidomide [13, 52, 167–169] improved when they were combined in triplets [51, 68, 78, 164, 170, 171]. Recent clinical studies with novel drugs have included triple-refractory patients, and aimed to improve outcomes in this population (Table 7) [94, 171]. Nevertheless, some questions remain, such as whether a single definition for double-refractoriness exists and whether this definition is applicable when new drugs shift to upfront use, and whether it is advisable to reuse in a new combination certain drugs to which the patient has developed refractoriness.

**Table 7:** Outcomes of double-refractory RRMM patients included in the main clinical trials

Study	Phase	Refractoriness	***N***	ORR (%)	≥VGPR/CRR/MRD (%)	Median PFS (months)	Median OS (months)	Median DoR (months)
Pd - MM-003 [52, 168]	3	V + *R*	225	28	6/-/-	3.7	11.1	7.0
Pd - MM-010 [13]	3	V + *R*	547	32.4	7.8/0.5/-	4.2	11.9	-
D - SIRIUS [14]	2	PI + IMiD	30	29.7	-	-	-	-
D - GEN501 + SIRIUS [169]	2	PI + IMiD	148*	30.4	14/5/-	4.0	20.5	8.0
IPd – ICARIA-MM [170]	3	PI + *R*	111	59	29.7/-/-	11.2	-	-
DPd – APOLLO [53]	3	PI + IMiD	64	-	-	7.7	-	-
DPd – MMY-1001 [68]	1	PI + IMiD	73	57.5	-	-	-	-
DKd – CANDOR [51, 164]	3	-	RR: 99 VR: 100	RR: 79.8 VR: 79.0	RR: -/-/13.1 VR: -/-/7.0	RR: NA VR: 14.2	-	-
DKd – MMY-1001 [171]	1	PI + IMiD	25	83	-/-/6.9	25.7	NA 1y-OS 75%	-
IKd – IKEMA [50]	3	-	RR: 57 VR: 52	-	RR: 66.7/38.6/24.6 VR: 55.8/28.8/17.3	RR: NA VR: NA	-	-
EloPd – ELOQUENT-3 [78]	2	PI + *R*	41	-	-	10.2	-	-
KPd – EMN07 [172]	1/2	V + *R*	21	71	24/5/-	10.3	-	-
Sd – STORM [65]	2b	PI + IMiD + D	122	26	6.56/1.64/-	3.7	8.6	4.4
Belamaf – DREAMM-2 [94]	2	PI + IMiD + D	97	32	18/7/-	2.8	13.7	11

*Patients from GEN-501 and SIRIUS trials who received daratumumab at 16 mg/kg are presented together. Not all are double refractory, but 87% are refractory to IMiD and PI

*Belamaf* belantamab mafodotin; *CRR* complete response rate; *D* daratumumab; *DKd*: daratumumab/carfolzimib/dexomethasone; *DoR* duration of response; *DPd;* daratumumab/pomalidomide/dexamethasone; *EloPd* elotuzumab/pomalidomide/dexamethasone; *IKd* isatuximab/carfilzomib/dexamethasone; *IMiD* immunomodulatory drug; *IPd* isatuximab/pomalidomide/dexamethasone; *Kd* carfilzomib/dexamethasone; *KPd* carfilzomib/pomalidomide/dexamethasone *L* lenalidomide; *LR* lenalidomide-refractory; *MRD* minimal residual disease; *NA* not achieved; *NR* not reached; *ORR* overall response rate; *OS* overall survival; *Pd* omalidomide/dexamethasone; *PFS* progression-free survival; *PI* proteasome

### Pros and cons of using the characteristics of MM as inclusion criteria in recent finalist phase 3 clinical trials for RRMM

Intense research into new agents for MM management has led to a wide range of possible drug combinations [173]. One of the goals at present, especially in the context of first relapse, is to obtain bone marrow and PET&MRD negativity, which are associated with better PFS and OS outcomes [174]. Another interesting positive aspect is the incorporation of immunotherapy, including CAR-T cells, as a new therapeutic approach to improve survival [175]. Finally, there is growing evidence that previous use of lenalidomide is unlikely to have an impact on response or survival if pomalidomide is used in subsequent therapies [39].

Some uncertainties in the management of RRMM patients should be also considered. Firstly, the proportion of upfront lenalidomide-treated patients is higher in the real-world (RW) than in the clinical trial setting [39]. This makes it difficult to transfer the results of RCTs to clinical practice, because a growing number of patients are currently receiving lenalidomide as part of their first-line treatment, and this differs greatly from the results of the main RRMM studies. Inclusion criteria is often vague, and efforts should be made to define these more clearly in upcoming phase 3 trials. There is also a need for a more accurate definition of the concept of progressive disease, a criterion that currently allows the inclusion of an unspecified percentage of patients in biological progression in most clinical studies, and of the duration of previous therapy as a prognostic and predictive factor of survival. It is also necessary to ensure the homogeneity of patient populations in RCTs as far as possible by defining, for example, the number of previous lines of therapy. Multi-refractoriness to multiple drugs used in MM is of particular concern. In this context, it is important to remember that survival to triple or higher refractoriness is probably shorter in the Revised International Staging System (R-ISS) II and III compared to stage I. In addition, little has been done to evaluate the transferability of clinical trial efficacy and safety results to the RW, and some results suggest the existence of a trial efficacy/RW effectiveness gap that limits the generalizability of clinical trial conclusions [176]. Finally, it is important to bear in mind that most RCTs do not include special populations (renal failure, extramedullary disease, etc.) that are frequently found in RRMM patients.

### RW patients vs. patients included in phase 3 trials for MM. Exclusion criteria bias

A growing body of evidence shows the gap between RRMM patients in RCTs and RW studies. Patients included in RCTs represent a select group of MM patients. Multiple confounding factors influence the interpretation of efficacy (RCTs) or effectiveness (RW), making these impossible to compare, and potentially affecting the external validity of the study. There are also differences in safety in RW settings *versus* RCTs, highlighting the importance of post marketing pharmacovigilance studies.

Older patients and patients with a high comorbidity burden are frequently underrepresented in RCTs. Renal impairment is a well-known prognostic factor and a commonly applied exclusion criterion in RCTs. As a result, PFS is generally shorter in RW studies compared to RCTs, and duration of therapy is reduced in RW due to poorer tolerability.

A recent RW study has shown that up to 75% of RRMM patients receiving routine care do not meet the eligibility criteria of hallmark RCTs in approved or recommended regimens in the RW setting. Moreover, OS was significantly worse (50% increased risk of mortality) in patients unable to meet study’s eligibility criteria. The most common reasons for RCT ineligibility were renal insufficiency and other malignancies [177]. In a retrospective study including 1601 RRMM patients, 40% received more than 2 lines of therapy. Substantial variation in RW PFS and OS was observed, ranging from 3.5-12.0 and 5.8-48.2 months, respectively. Overall, these values are lower than those observed in recent RCTs for the same agents in third and higher lines of therapy [178].

Translating RCTs findings to the RW setting is challenging. The stricter the RCT inclusion/exclusion criteria, the greater difference in results in RW studies. However, little or no gap is observed when all-oral regimens or bortezomib-based regimens are used. The discrepancy between RW and RCTs data is also minimized in later *versus* earlier lines of therapy [179].

Both RCTs and RW studies provide complementary information of paramount importance in clinical decision-making. RW effectiveness is increasingly emphasized when determining the effectiveness of new approved regimens in the heterogeneous and complex population of RRMM patients. In this regard, it is essential to have access to high quality RW data provided by consolidated population-based cancer registries (PBCRs). In coming years, therefore, RCTs and PBCRs will need to work closely together at the local, regional and national level.

## Final considerations: key points in the orientation of RRMM treatment

The therapeutic strategy for RRMM must follow a comprehensive, standardized, personalized approach that includes patient, disease, biology and previous therapy (response characteristics and toxicity). The treatment of RRMM is a rapidly changing field, and it is highly recommended to encourage patients to participate in a clinical study, if available. The best regimen should be chosen, taking into consideration all clinically relevant variables, as well as patient preference, the cost-benefit ratio, and local availability.

Achieving the deepest possible response must always be weighed up against achieving the best quality of life, particularly in the elderly, frail population. The importance of supportive care throughout RRMM management must be emphasized. Several phase 3 RCTs have shown, in most cases, the superiority of triplets over the doublet Vd and Rd, and later, Kd and Pd. Determining patient refractoriness to these agents at the time of relapse is a key factor in the choice of the optimal regimen.

Approaches to the treatment of triple-class (PIs, IMiDs and monoclonal antibodies) RRMM patients, including CC, sASCT and retreatment, are currently limited. CC is still a therapeutic option (almost always associated with new agents), and is usually used as a bridge to more definitive treatment, or in certain circumstances such as bulky disease or neurological complications that require rapid cytoreduction. High dose therapy followed by sASCT remains a safe and probably cost-effective approach for a selected subgroup of RRMM patients. However, continuous approval of new agents and the emergence of safer and more effective combinations have now called the role of sASCT into question. All in all, more evidence is needed before a paradigm shift occurs.

Retreatment has been used with limited results when no other choice is available, but new drugs should now be used instead whenever possible. New combinations based on second generation PIs and IMiDs, new monoclonal antibodies, histone deacetylase inhibitors, new class drugs such as venetoclax in the presence of t(11;14) or selinexor, and drugs with new mechanisms of action such as melflufen can be suggested after approval.

Research should prioritize triple-class refractory patients, and they should be encouraged to participate in clinical trials. BCMA-directed CAR-T-cell therapy, bispecific antibodies and belantamab mafodotin are new immunotherapeutic approaches that have shown promising results. Non-myeloablative reduced-intensity conditioning allogeneic stem cell transplantation (RIC Allo) could be considered in certain young patients with high-risk cytogenetics, particularly in the context of a clinical study.

Translating the efficacy results of RCTs into RW effectiveness is challenging, mainly due to differences in clinical characteristics in both populations and different levels of tolerability, particularly for non-oral drugs. Comparison between most recent phase 3 RCTs and RW studies confirm the existence of an important gap, with the exception of studies using all-oral regimens and V-based regimens. Currently, about half of all patients cannot be included in RCTs due to comorbidities (mainly renal failure) or special features (non-secretory myeloma, plasma cell leukemia, extramedullary disease, etc.).

## Conclusions

The dramatic advances made in biology, prognosis and therapy for RRMM patients in recent years has created new challenges:

- How to order the sequence of lines of treatment of RRMM after the incorporation of IMiDs and AntiCD38 MoAbs into the first-line approach?.
- Is there still a role for chemotherapy in the treatment of RRMM patients?
- Should ASCT be abandoned following the emergence of new biological and cellular therapies? In this regard, more evidence on the optimal timing of CAR-T cell therapy is needed.
- In this new COVID-19 era, which is the best approach for the treatment of patients with MM? [180].
- Is maintenance therapy until progression or intolerance still the most appropriate strategy for improving survival?

Despite the exciting therapeutic development of many new generation agents and combination regimens (including IMiDs or immunotherapy agents, as anti-CD38 monoclonal antibodies, conjugated antibodies, bispecific antibodies, and CAR-T) that are providing considerable improved progression-free survival and overall survival, there is still room left to further increase the rates of response and survival. RRMM management will soon be improved by the introduction of new biomarkers that will improve patient stratification and prognosis. This along with the definition of newer treatment algorithms that allow clinicians to design personalized therapeutic regimens (which balance the clinical and biological characteristics of MM, and patients’ comorbidities and preferences) will likely result in a safer and more effective precision medicine [181].

## Data Availability

Not applicable

## Abbreviations

ASCT/sASCT: Autologous stem cells transplants/ salvage autologous stem cells transplants
CC: Conventional chemotherapy
DKd: Daratumumab plus carfilzomib plus dexamethasone
DPd: Daratumumab plus pomalidomide plus dexamethasone
DRd: Daratumumab plus lenalidomide plus dexamethasone
DVd: Daratumumab plus bortezomib plus dexamethasone
HR: Hazard ratio
IMiDs: Immunomodulatory drugs
IRRs: Infusion-related reactions
Isa-Kd: Isatuximab plus carfilzomib plus dexamethasone
Isa-Pd: Isatuximab plus pomalidomide plus dexamethasone
Isa-Rd: Isatuximab plus lenalidomide plus dexamethasone
Kd: Carfilzomib plus dexamethasone
KPd: Carfilzomib plus pomalidomide plus dexamethasone
KRd: Carfilzomib plus lenalidomide plus dexamethasone
MoAbs: Monoclonal antibodies
MM: Multiple myeloma
OS: Overall survival
ORR: Overall response rate
PFS: Progression-free survival
Pd: Pomalidomide plus dexamethasone
PIs: Proteasome inhibitors
PVd: Pomalidomide plus bortezomib plus dexamethasone
RCTs: Randomized clinical trials
Rd: Lenalidomide plus dexamethasone
RR: Relapsed/refractory
RW: Real world
Vd: Bortezomib plus dexamethasone
VRd: Bortezomib plus lenalidomide plus dexamethasone
VTd: Bortezomib plus thalidomide plus dexamethasone
XPO inhibitor: inhibitor of nuclear exportin

## References

[1] Dimopoulos MA, Moreau P, Terpos E, Mateos MV, Zweegman S, Cook G (2021). Multiple Myeloma: EHA-ESMO Clinical Practice Guidelines for Diagnosis, Treatment and Follow-up. Hemasphere.

[2] Gulla A, Anderson KC (2020). Multiple myeloma: the (r) evolution of current therapy and a glance into future. Haematologica.

[3] Durer C, Durer S, Lee S, Chakraborty R, Malik MN, Rafae A (2020). Treatment of relapsed multiple myeloma: Evidence-based recommendations. Blood Rev.

[4] Barlogie B, Smith L, Alexanian R (1984). Effective treatment of advanced multiple myeloma refractory to alkylating agents. N Engl J Med.

[5] Cook G, Ashcroft AJ, Cairns DA, Williams CD, Brown JM, Cavenagh JD (2016). The effect of salvage autologous stem-cell transplantation on overall survival in patients with relapsed multiple myeloma (final results from BSBMT/UKMF Myeloma X Relapse [Intensive]): a randomised, open-label, phase 3 trial. Lancet Haematol.

[6] Cavo M, Terpos E, Bargay J, Einsele H, Cavet J, Greil R (2018). The multiple myeloma treatment landscape: international guideline recommendations and clinical practice in Europe. (1747-4094 (Electronic)). Expert Rev Hematol.

[7] Attal M, Lauwers-Cances V, Hulin C, Leleu X, Caillot D, Escoffre M (2017). Lenalidomide, bortezomib, and dexamethasone with transplantation for myeloma. N Engl J Med.

[8] Rosiñol L, Oriol A, Teruel AI, Hernández D, López-Jiménez J, de la Rubia J (2012). Superiority of bortezomib, thalidomide, and dexamethasone (VTD) as induction pretransplantation therapy in multiple myeloma: a randomized phase 3 PETHEMA/GEM study. Blood.

[9] Mateos MV, Cavo M, Blade J, Dimopoulos MA, Suzuki K, Jakubowiak A (2020). Overall survival with daratumumab, bortezomib, melphalan, and prednisone in newly diagnosed multiple myeloma (ALCYONE): a randomised, open-label, phase 3 trial. Lancet.

[10] Attal M, Richardson PG, Rajkumar SV, San-Miguel J, Beksac M, Spicka I (2019). Isatuximab plus pomalidomide and low-dose dexamethasone versus pomalidomide and low-dose dexamethasone in patients with relapsed and refractory multiple myeloma (ICARIA-MM): a randomised, multicentre, open-label, phase 3 study. Lancet.

[11] Dimopoulos MA, Moreau P, Palumbo A, Joshua D, Pour L, Hájek R (2016). Carfilzomib and dexamethasone versus bortezomib and dexamethasone for patients with relapsed or refractory multiple myeloma (ENDEAVOR): a randomised, phase 3, open-label, multicentre study. Lancet Oncol.

[12] Dimopoulos MA, Oriol A, Nahi H, San-Miguel J, Bahlis NJ, Usmani SZ (2016). *Daratumumab, Lenalidomide, and Dexamethasone for Multiple Myeloma*. N Engl J Med.

[13] Dimopoulos MA, Palumbo A, Corradini P, Cavo M, Delforge M, Di Raimondo F (2016). Safety and efficacy of pomalidomide plus low-dose dexamethasone in STRATUS (MM-010): a phase 3b study in refractory multiple myeloma. Blood.

[14] Lonial S, Dimopoulos M, Palumbo A, White D, Grosicki S, Spicka I (2015). Elotuzumab Therapy for Relapsed or Refractory Multiple Myeloma. N Engl J Med.

[15] Moreau P, Masszi T, Grzasko N, Bahlis NJ, Hansson M, Pour L (2016). Oral Ixazomib, Lenalidomide, and Dexamethasone for Multiple Myeloma. N Engl J Med.

[16] Moreau P, Mateos MV, Berenson JR, Weisel K, Lazzaro A, Song K (2018). Once weekly versus twice weekly carfilzomib dosing in patients with relapsed and refractory multiple myeloma (A.R.R.O.W.): interim analysis results of a randomised, phase 3 study. Lancet Oncol.

[17] Palumbo A, Chanan-Khan A, Weisel K, Nooka AK, Masszi T, Beksac M (2016). Daratumumab, Bortezomib, and Dexamethasone for Multiple Myeloma. N Engl J Med.

[18] San-Miguel JF, Hungria VT, Yoon SS, Beksac M, Dimopoulos MA, Elghandour A (2014). Panobinostat plus bortezomib and dexamethasone versus placebo plus bortezomib and dexamethasone in patients with relapsed or relapsed and refractory multiple myeloma: a multicentre, randomised, double-blind phase 3 trial. Lancet Oncol.

[19] Stewart AK, Rajkumar SV, Dimopoulos MA, Masszi T, Špička I, Oriol A (2015). Carfilzomib, lenalidomide, and dexamethasone for relapsed multiple myeloma. N Engl J Med.

[20] Durie BG, Harousseau JL, Miguel JS, Bladé J, Barlogie B, Anderson K (2006). International uniform response criteria for multiple myeloma. Leukemia.

[21] van de Donk N, Pawlyn C, Yong KL (2021). Multiple myeloma. Lancet.

[22] Afram G, Gran C, Borg Bruchfeld J, Wagner AK, Hussain A, Alici E (2020). Impact of performance status on overall survival in patients with relapsed and/or refractory multiple myeloma: Real-life outcomes of daratumumab treatment. Eur J Haematol.

[23] Giri S, Huntington SF, Wang R, Zeidan AM, Podoltsev N, Gore SD (2020). Chromosome 1 abnormalities and survival of patients with multiple myeloma in the era of novel agents. Blood Adv.

[24] Kumar SK, Therneau TM, Gertz MA, Lacy MQ, Dispenzieri A, Rajkumar SV (2004). Clinical course of patients with relapsed multiple myeloma. Mayo Clin Proc.

[25] Vogl DT, Stadtmauer EA, Richardson PG, Sonneveld P, Schuster MW, Irwin D (2009). Impact of prior therapies on the relative efficacy of bortezomib compared with dexamethasone in patients with relapsed/refractory multiple myeloma. Br J Haematol.

[26] Wang M, Dimopoulos MA, Chen C, Cibeira MT, Attal M, Spencer A (2008). Lenalidomide plus dexamethasone is more effective than dexamethasone alone in patients with relapsed or refractory multiple myeloma regardless of prior thalidomide exposure. Blood.

[27] Kunacheewa C, Orlowski RZ (2019). New Drugs in Multiple Myeloma. Annu Rev Med.

[28] Fosså A, Muer M, Kasper C, Welt A, Seeber S, Nowrousian MR (1998). Bolus vincristine and epirubicin with cyclophosphamide and dexamethasone (VECD) as induction and salvage treatment in multiple myeloma. Leukemia.

[29] Lee CK, Barlogie B, Zangari M, Fassas A, Anaissie E, Morris C (2002). Transplantation as salvage therapy for high-risk patients with myeloma in relapse. Bone Marrow Transplant.

[30] Mo CC, Richardson PG (2020). Pomalidomide in lenalidomide-refractory multiple myeloma: Far from futile. Br J Haematol.

[31] Richardson PG, Oriol A, Beksac M, Liberati AM, Galli M, Schjesvold F (2019). Pomalidomide, bortezomib, and dexamethasone for patients with relapsed or refractory multiple myeloma previously treated with lenalidomide (OPTIMISMM): a randomised, open-label, phase 3 trial. Lancet Oncol.

[32] Griffin PT, Ho VQ, Fulp W, Nishihori T, Shain KH, Alsina M (2015). A comparison of salvage infusional chemotherapy regimens for recurrent/refractory multiple myeloma. Cancer.

[33] Lakshman A, Singh PP, Rajkumar SV, Dispenzieri A, Lacy MQ, Gertz MA (2018). Efficacy of VDT PACE-like regimens in treatment of relapsed/refractory multiple myeloma. Am J Hematol.

[34] Lenhoff S, Hjorth M, Turesson I, Westin J, Gimsing P, Wislöff F (2006). Intensive therapy for multiple myeloma in patients younger than 60 years. Long-term results focusing on the effect of the degree of response on survival and relapse pattern after transplantation. Haematologica.

[35] Alvares CL, Davies FE, Horton C, Patel G, Powles R, Morgan GJ (2006). The role of second autografts in the management of myeloma at first relapse. Haematologica.

[36] Qazilbash MH, Saliba R, De Lima M, Hosing C, Couriel D, Aleman A (2006). Second autologous or allogeneic transplantation after the failure of first autograft in patients with multiple myeloma. Cancer.

[37] Paccagnella A, Chiarion-Sileni V, Soesan M, Baggio G, Bolzonella S, De Besi P (1991). Second and third responses to the same induction regimen in relapsing patients with multiple myeloma. Cancer.

[38] Palumbo A, Bringhen S, Falco P, Cavallo F, Ambrosini MT, Avonto I (2007). Time to first disease progression, but not beta2-microglobulin, predicts outcome in myeloma patients who receive thalidomide as salvage therapy. Cancer.

[39] Kastritis E, Roussou M, Gavriatopoulou M, Kanellias N, Migkou M, Eleutherakis-Papaiakovou E (2019). Impact of last lenalidomide dose, duration, and IMiD-free interval in patients with myeloma treated with pomalidomide/dexamethasone. Blood Adv.

[40] Kumar SK, Dispenzieri A, Fraser R, Mingwei F, Akpek G, Cornell R (2018). Early relapse after autologous hematopoietic cell transplantation remains a poor prognostic factor in multiple myeloma but outcomes have improved over time. Leukemia.

[41] Mateos MV, Martínez-López J, Hernández MT, Ocio EM, Rosiñol L, Martínez R (2016). Sequential vs alternating administration of VMP and Rd in elderly patients with newly diagnosed MM. Blood.

[42] Kumar S, Paiva B, Anderson KC, Durie B, Landgren O, Moreau P (2016). International Myeloma Working Group consensus criteria for response and minimal residual disease assessment in multiple myeloma. Lancet Oncol.

[43] Bladé J, Samson D, Reece D, Apperley J, Björkstrand B, Gahrton G (1998). Criteria for evaluating disease response and progression in patients with multiple myeloma treated by high-dose therapy and haemopoietic stem cell transplantation. Myeloma Subcommittee of the EBMT. European Group for Blood and Marrow Transplant. Br J Haematol.

[44] Paiva B, Puig N, Cedena MT, Rosiñol L, Cordón L, Vidriales MB (2020). Measurable Residual Disease by Next-Generation Flow Cytometry in Multiple Myeloma. J Clin Oncol.

[45] Hillengass J, Usmani S, Rajkumar SV, Durie BGM, Mateos MV, Lonial S (2019). International myeloma working group consensus recommendations on imaging in monoclonal plasma cell disorders. Lancet Oncol.

[46] Deighan WI, Winton VJ, Melani RD, Anderson LC, McGee JP, Schachner LF, et al. Development of novel methods for non-canonical myeloma protein analysis with an innovative adaptation of immunofixation electrophoresis, native top-down mass spectrometry, and middle-down de novo sequencing. Clin Chem Lab Med. 2020. 10.1515/cclm-2020-1072 PMC8055720 33079696

[47] Jackson GH, Davies FE, Pawlyn C, Cairns DA, Striha A, Collett C (2019). Response-adapted intensification with cyclophosphamide, bortezomib, and dexamethasone versus no intensification in patients with newly diagnosed multiple myeloma (Myeloma XI): a multicentre, open-label, randomised, phase 3 trial. Lancet Haematol.

[48] Moreau P, Siegel DS, Goldschmidt H, Niesvizky R, Bringhen S, Orlowski RZ (2018). Subgroup Analysis of Patients with Biochemical or Symptomatic Relapse at the Time of Enrollment in the Endeavor Study. Blood.

[49] Alexanian R, Balcerzak S, Bonnet JD, Gehan EA, Haut A, Hewlett JS (1975). Prognostic factors in multiple myeloma. Cancer.

[50] Moreau P., Dimopoulos M.A., Mikhael J., Yong K., Capra M., Facon T., et al., Isatuximab plus carfilzomib and dexamethasone vs carfilzomib and dexamethasone in relapsed/refractory multiple myeloma (IKEMA): Interim analysis of a phase 3, randomized, open-label study, in The 25th European Hematology Association Annual Congress (EHA25 Virtual). 2020.

[51] Dimopoulos M, Quach H, Mateos MV, Landgren O, Leleu X, Siegel D (2020). Carfilzomib, dexamethasone, and daratumumab versus carfilzomib and dexamethasone for patients with relapsed or refractory multiple myeloma (CANDOR): results from a randomised, multicentre, open-label, phase 3 study. Lancet.

[52] Miguel JS, Weisel K, Moreau P, Lacy M, Song K, Delforge M (2013). Pomalidomide plus low-dose dexamethasone versus high-dose dexamethasone alone for patients with relapsed and refractory multiple myeloma (MM-003): a randomised, open-label, phase 3 trial. Lancet Oncol.

[53] Dimopoulos MA, Terpos E, Boccadoro M, Delimpasi S, Beksac M, Katodritou E (2021). Daratumumab plus pomalidomide and dexamethasone versus pomalidomide and dexamethasone alone in previously treated multiple myeloma (APOLLO): an open-label, randomised, phase 3 trial. Lancet Oncol.

[54] Spencer A, Lentzsch S, Weisel K, Avet-Loiseau H, Mark TM, Spicka I (2018). Daratumumab plus bortezomib and dexamethasone versus bortezomib and dexamethasone in relapsed or refractory multiple myeloma: updated analysis of CASTOR. Haematologica.

[55] Dimopoulos MA, Lonial S, Betts KA, Chen C, Zichlin ML, Brun A (2018). Elotuzumab plus lenalidomide and dexamethasone in relapsed/refractory multiple myeloma: Extended 4-year follow-up and analysis of relative progression-free survival from the randomized ELOQUENT-2 trial. Cancer.

[56] Mateos MV, Blacklock H, Schjesvold F, Oriol A, Simpson D, George A (2019). Pembrolizumab plus pomalidomide and dexamethasone for patients with relapsed or refractory multiple myeloma (KEYNOTE-183): a randomised, open-label, phase 3 trial. Lancet Haematol.

[57] Rajkumar SV (2020). Multiple myeloma: 2020 update on diagnosis, risk-stratification and management. Am J Hematol.

[58] Dimopoulos MA, Stewart AK, Masszi T, Špička I, Oriol A, Hájek R (2017). Carfilzomib-lenalidomide-dexamethasone vs lenalidomide-dexamethasone in relapsed multiple myeloma by previous treatment. Blood Cancer J.

[59] Siegel DS, Dimopoulos MA, Ludwig H, Facon T, Goldschmidt H, Jakubowiak A (2018). Improvement in Overall Survival With Carfilzomib, Lenalidomide, and Dexamethasone in Patients With Relapsed or Refractory Multiple Myeloma. J Clin Oncol.

[60] Jakubowiak A, Rosenbaum C, Stephens L, Kukreti V, Cole C, Zimmerman T, et al. Final results of phase (ph) 1/2 study of carfilzomib, pomalidomide, and dexamethasone (KPD) in patients (pts) with relapsed/refractory multiple myeloma (RRMM): a multi-center MMRC study, in The 22th Congress of the European Hematology Association. Madrid, Spain; 2017.

[61] Rosenbaum CA, Stephens LA, Kukreti V, Zonder JA, Cole C, Zimmerman TM (2016). Phase 1/2 study of carfilzomib, pomalidomide, and dexamethasone (KPd) in patients (Pts) with relapsed/refractory multiple myeloma (RRMM): A Multiple Myeloma Research Consortium multicenter study. J Clin Oncol.

[62] Shah JJ, Stadtmauer EA, Abonour R, Cohen AD, Bensinger W, Gasparetto C (2013). Phase I/II Dose Expansion Of a Multi-Center Trial Of Carfilzomib and Pomalidomide With Dexamethasone (Car-Pom-d) In Patients With Relapsed/Refractory Multiple Myeloma. Blood.

[63] Sonneveld P, Terpos E, Dimopoulos MA, Ukropec J, Smith E, Houkes N (2018). Pomalidomide and dexamethasone (pom-dex) with or without daratumumab (DARA) in patients (pts) with relapsed or refractory multiple myeloma (RRMM): A multicenter, randomized, phase 3 study (APOLLO). J Clin Oncol.

[64] Mushtaq A, Iftikhar A, Hassan H, Lakhani M, Sagar F, Kamal A (2019). Pomalidomide-Based Regimens for Treatment of Relapsed and Relapsed/Refractory Multiple Myeloma: Systematic Review and Meta-analysis of Phase 2 and 3 Clinical Trials. Clin Lymphoma Myeloma Leuk.

[65] Chari A, Vogl DT, Gavriatopoulou M, Nooka AK, Yee AJ, Huff CA (2019). Oral Selinexor-Dexamethasone for Triple-Class Refractory Multiple Myeloma. N Engl J Med.

[66] Mateos MV, Masszi T, Grzasko N, Hansson M, Sandhu I, Pour L (2017). Impact of prior therapy on the efficacy and safety of oral ixazomib-lenalidomide-dexamethasone vs. placebo-lenalidomide-dexamethasone in patients with relapsed/refractory multiple myeloma in TOURMALINE-MM1. Haematologica.

[67] Richardson PG, Attal M, Rajkumar SV, San Miguel J, Beksac M, Spicka I (2019). A phase III randomized, open label, multicenter study comparing isatuximab, pomalidomide, and low-dose dexamethasone versus pomalidomide and low-dose dexamethasone in patients with relapsed/refractory multiple myeloma (RRMM). J Clin Oncol.

[68] Chari A, Suvannasankha A, Fay JW, Arnulf B, Kaufman JL, Ifthikharuddin JJ (2017). Daratumumab plus pomalidomide and dexamethasone in relapsed and/or refractory multiple myeloma. Blood.

[69] van de Donk N, Richardson PG, Malavasi F (2018). CD38 antibodies in multiple myeloma: back to the future. Blood.

[70] Moreno L, Perez C, Zabaleta A, Manrique I, Alignani D, Ajona D (2019). The Mechanism of Action of the Anti-CD38 Monoclonal Antibody Isatuximab in Multiple Myeloma. Clin Cancer Res.

[71] European Commission approves Sarclisa® (isatuximab) for adults with relapsed and refractory multiple myeloma. Published June 2, 2020. https://bit.ly/2U6kxpD. Accessed June 2, 2020.

[72] Mateos MV, Sonneveld P, Hungria V, Nooka AK, Estell JA, Barreto W (2020). Daratumumab, Bortezomib, and Dexamethasone Versus Bortezomib and Dexamethasone in Patients With Previously Treated Multiple Myeloma: Three-year Follow-up of CASTOR. Clin Lymphoma Myeloma Leuk.

[73] Bahlis NJ, Dimopoulos MA, White DJ, Benboubker L, Cook G, Leiba M (2020). Daratumumab plus lenalidomide and dexamethasone in relapsed/refractory multiple myeloma: extended follow-up of POLLUX, a randomized, open-label, phase 3 study. Leukemia.

[74] Kaufman J, Usmani S, San-Miguel J, Bahlis N, White D, Benboubker L (2019). Four-Year Follow-up of the Phase 3 Pollux Study of Daratumumab Plus Lenalidomide and Dexamethasone (D-Rd) Versus Lenalidomide and Dexamethasone (Rd) Alone in Relapsed or Refractory Multiple Myeloma (RRMM). Blood.

[75] van Beurden-Tan CHY, Franken MG, Blommestein HM, Uyl-de Groot CA, Sonneveld P (2017). Systematic Literature Review and Network Meta-Analysis of Treatment Outcomes in Relapsed and/or Refractory Multiple Myeloma. J Clin Oncol.

[76] Mateos MV, Nahi H, Legiec W, Grosicki S, Vorobyev V, Spicka I (2020). Subcutaneous versus intravenous daratumumab in patients with relapsed or refractory multiple myeloma (COLUMBA): a multicentre, open-label, non-inferiority, randomised, phase 3 trial. Lancet Haematol.

[77] Raab MS, Engelhardt M, Blank A, Goldschmidt H, Agis H, Blau IW (2020). MOR202, a novel anti-CD38 monoclonal antibody, in patients with relapsed or refractory multiple myeloma: a first-in-human, multicentre, phase 1-2a trial. Lancet Haematol.

[78] Dimopoulos MA, Dytfeld D, Grosicki S, Moreau P, Takezako N, Hori M (2018). Elotuzumab plus Pomalidomide and Dexamethasone for Multiple Myeloma. N Engl J Med.

[79] Richardson PG, Bringhen S, Voorhees P, Plesner T, Mellqvist UH, Reeves B (2020). Melflufen plus dexamethasone in relapsed and refractory multiple myeloma (O-12-M1): a multicentre, international, open-label, phase 1-2 study. Lancet Haematol.

[80] Grosicki S, Simonova M, Spicka I, Pour L, Kriachok I, Gavriatopoulou M (2020). Once-per-week selinexor, bortezomib, and dexamethasone versus twice-per-week bortezomib and dexamethasone in patients with multiple myeloma (BOSTON): a randomised, open-label, phase 3 trial. Lancet.

[81] Ocio EM, Efebera YA, Granell M, Hajek R, Maisnar V, Karlin L (2019). ANCHOR (OP-104): Updated Efficacy and Safety from a Phase 1/2 Study of Melflufen and Dexamethasone Plus Bortezomib or Daratumumab in Patients with Relapsed/Refractory Multiple Myeloma (RRMM) Refractory to an IMiD or a Proteasome Inhibitor (PI). Blood.

[82] Basali D, Chakraborty R, Rybicki L, Rosko N, Reed J, Karam M (2020). Real-world data on safety and efficacy of venetoclax-based regimens in relapsed/refractory t(11;14) multiple myeloma. Br J Haematol.

[83] Kumar S, Harrison SJ, Cavo M, Rubia JdL, Popat R, Gasparetto C (2020). Updated results from BELLINI, a phase III study of venetoclax or placebo in combination with bortezomib and dexamethasone in relapsed/refractory multiple myeloma. J Clin Oncol.

[84] Bahlis NJ, Sutherland H, White D, Sebag M, Lentzsch S, Kotb R (2018). Selinexor plus low-dose bortezomib and dexamethasone for patients with relapsed or refractory multiple myeloma. Blood.

[85] San-Miguel JF, Hungria VT, Yoon SS, Beksac M, Dimopoulos MA, Elghandour A (2016). Overall survival of patients with relapsed multiple myeloma treated with panobinostat or placebo plus bortezomib and dexamethasone (the PANORAMA 1 trial): a randomised, placebo-controlled, phase 3 trial. Lancet Haematol.

[86] Bertamini L, Bonello F, Boccadoro M, Bringhen S (2020). New drugs in early development for treating multiple myeloma: all that glitters is not gold. Expert Opin Investig Drugs.

[87] Bjorklund CC, Kang J, Amatangelo M, Polonskaia A, Katz M, Chiu H (2020). Iberdomide (CC-220) is a potent cereblon E3 ligase modulator with antitumor and immunostimulatory activities in lenalidomide- and pomalidomide-resistant multiple myeloma cells with dysregulated CRBN. Leukemia.

[88] Wong L, Jiménez Nuñez MD, Bahlis NJ, Vangsted AJ, Ramasamy K, Trudel S (2020). Pharmacodynamic (PD) responses drive dose/schedule selection of CC-92480, a novel CELMoD agent, in a phase 1 dose-escalation study in relapsed/refractory multiple myeloma (RRMM). J Clin Oncol.

[89] Lonial S, Van de Donk N, Popat R, Zonder JA, Minnema M, Larsen JT (2019). A Phase 1b/2a Study of the CELMoD Iberdomide (CC-220) in Combination With Dexamethasone in Patients with Relapsed/Refractory Multiple Myeloma. Clin Lymphoma Myeloma Leuk.

[90] van de Donk NWCJ, Popat R, Larsen J, Minnema MC, Jagannath S, Oriol A (2020). First Results of Iberdomide (IBER; CC-220) in Combination with Dexamethasone (DEX) and Daratumumab (DARA) or Bortezomib (BORT) in Patients with Relapsed/Refractory Multiple Myeloma (RRMM). Blood.

[91] Richardson PG, Vangsted AJ, Ramasamy K, Trudel S, Martínez J, Mateos MV (2020). First-in-human Phase 1 study of the novel celmod agent CC-92480 combined with dexamethasone in patients with relapsed/refractory multiple myeloma, in The 25th European Hematology Association Annual Congress (EHA25 Virtual).

[92] Gandhi UH, Cornell RF, Lakshman A, Gahvari ZJ, McGehee E, Jagosky MH (2019). Outcomes of patients with multiple myeloma refractory to CD38-targeted monoclonal antibody therapy. Leukemia.

[93] Shah N, Chari A, Scott E, Mezzi K, Usmani SZ (2020). B-cell maturation antigen (BCMA) in multiple myeloma: rationale for targeting and current therapeutic approaches. Leukemia.

[94] Lonial S, Lee HC, Badros A, Trudel S, Nooka AK, Chari A (2020). Belantamab mafodotin for relapsed or refractory multiple myeloma (DREAMM-2): a two-arm, randomised, open-label, phase 2 study. Lancet Oncol.

[95] Nooka AK, Mateos Manteca MV, Bahlis N, Weisel K, Oriol A, Alonso Alonso A (2020). DREAMM-4: Evaluating safety and clinical activity of belantamab mafodotin in combination with pembrolizumab in patients with relapsed/refractory multiple myeloma (RRMM), in The 25th European Hematology Association Annual Congress (EHA25 Virtual).

[96] Popat R, Stockerl-Goldstein K, Quach H, Forbes A, Mateos MV, Khot A (2020). DREAMM-6: Safety and tolerability of belantamab mafodotin in combination with bortezomib/dexamethasone in relapsed/refractory multiple myeloma (RRMM), in The 25th European Hematology Association Annual Congress (EHA25 Virtual).

[97] Munshi NC, Anderson LD, Shah N, Madduri D, Berdeja J, Lonial S (2021). Idecabtagene Vicleucel in Relapsed and Refractory Multiple Myeloma. N Engl J Med.

[98] Mailankody S, Ghosh A, Staehr M, Purdon TJ, Roshal M, Halton E (2018). Clinical Responses and Pharmacokinetics of MCARH171, a Human-Derived Bcma Targeted CAR T Cell Therapy in Relapsed/Refractory Multiple Myeloma: Final Results of a Phase I Clinical Trial. Blood.

[99] Madduri D, Berdeja JG, Usmani SZ, Jakubowiak A, Agha M, Cohen AD (2020). CARTITUDE-1: Phase 1b/2 Study of Ciltacabtagene Autoleucel, a B-Cell Maturation Antigen-Directed Chimeric Antigen Receptor T Cell Therapy, in Relapsed/Refractory Multiple Myeloma. Blood.

[100] Costello CL, Cohen AD, Patel KK, Ali SS, Berdeja JG, Shah N (2020). Phase 1/2 Study of the Safety and Response of P-BCMA-101 CAR-T Cells in Patients with Relapsed/Refractory (r/r) Multiple Myeloma (MM) (PRIME) with Novel Therapeutic Strategies. Blood.

[101] Kumar SK, Baz RC, Orlowski RZ, Anderson LD, Ma H, Shrewsbury A (2020). Results from Lummicar-2: A Phase 1b/2 Study of Fully Human B-Cell Maturation Antigen-Specific CAR T Cells (CT053) in Patients with Relapsed and/or Refractory Multiple Myeloma. Blood.

[102] Brudno JN, Maric I, Hartman SD, Rose JJ, Wang M, Lam N (2018). T Cells Genetically Modified to Express an Anti–B-Cell Maturation Antigen Chimeric Antigen Receptor Cause Remissions of Poor-Prognosis Relapsed Multiple Myeloma. J Clin Oncol.

[103] Cohen AD, Garfall AL, Stadtmauer EA, Melenhorst JJ, Lacey SF, Lancaster E (2019). B cell maturation antigen-specific CAR T cells are clinically active in multiple myeloma. J Clin Invest.

[104] Alsina M., Shah N., Raje N. S., Jagannath S., Madduri D., Kaufman J. L., et al., Updated Results from the Phase I CRB-402 Study of Anti-Bcma CAR-T Cell Therapy bb21217 in Patients with Relapsed and Refractory Multiple Myeloma: Correlation of Expansion and Duration of Response with T Cell Phenotypes, in 62nd ASH Annual Meeting and Exposition (Online). 2020: O.

[105] Green DJ, Pont M, Sather BD, Cowan AJ, Turtle CJ, Till BG, et al. Fully Human Bcma Targeted Chimeric Antigen Receptor T Cells Administered in a Defined Composition Demonstrate Potency at Low Doses in Advanced Stage High Risk Multiple Myeloma. Blood. 2018;(132, Supplement 1):1011.

[106] Liu Y, Chen Z, Fang H, Wei R, Yu K, Jiang S (2018). Durable Remission Achieved from Bcma-Directed CAR-T Therapy Against Relapsed or Refractory Multiple Myeloma. Blood.

[107] Mikkilineni L, Manasanch E, Lam N, Vanasse D, Brudno J, Maric I (2019). T Cells Expressing an Anti-B-Cell Maturation Antigen (BCMA) Chimeric Antigen Receptor with a Fully-Human Heavy-Chain-Only Antigen Recognition Domain Induce Remissions in Patients with Relapsed Multiple Myeloma. Blood.

[108] Li C, Wang J, Wang D, Hu G, Yang Y, Zhou X (2019). Efficacy and Safety of Fully Human Bcma Targeting CAR T Cell Therapy in Relapsed/Refractory Multiple Myeloma. Blood.

[109] An G, Sui W, Wang T, Qu X, Zhang X, Yang J, et al. An Anti-Bcma CAR T-Cell Therapy (C-CAR088) Shows Promising Safety and Efficacy Profile in Relapsed or Refractory Multiple Myeloma. Blood. 2020;(136, Supplement 1):29–30.

[110] Mailankody S, Matous JV, Liedtke M, Sidana S, Malik S, Nath R (2020). Universal: An Allogeneic First-in-Human Study of the Anti-Bcma ALLO-715 and the Anti-CD52 ALLO-647 in Relapsed/Refractory Multiple Myeloma. Blood.

[111] Berdeja JG, Madduri D, Usmani SZ, Singh I, Zudaire E, Yeh T-M (2020). Update of CARTITUDE-1: A phase Ib/II study of JNJ-4528, a B-cell maturation antigen (BCMA)-directed CAR-T-cell therapy, in relapsed/refractory multiple myeloma. J Clin Oncol.

[112] Mailankody S, Jakubowiak AJ, Htut M, Costa LJ, Lee K, Ganguly S (2020). Orvacabtagene autoleucel (orva-cel), a B-cell maturation antigen (BCMA)-directed CAR T cell therapy for patients (pts) with relapsed/refractory multiple myeloma (RRMM): update of the phase 1/2 EVOLVE study (NCT03430011). J Clin Oncol.

[113] Cowan AJ, Pont M, Sather BD, Turtle CJ, Till BG, Nagengast AM (2019). Efficacy and Safety of Fully Human Bcma CAR T Cells in Combination with a Gamma Secretase Inhibitor to Increase Bcma Surface Expression in Patients with Relapsed or Refractory Multiple Myeloma. Blood.

[114] Lam N, Trinklein ND, Buelow B, Patterson GH, Ojha N, Kochenderfer JN (2020). Anti-BCMA chimeric antigen receptors with fully human heavy-chain-only antigen recognition domains. Nat Commun.

[115] Shah N, Alsina M, Siegel DS, Jagannath S, Madduri D, Kaufman JL (2018). Initial Results from a Phase 1 Clinical Study of bb21217, a Next-Generation Anti Bcma CAR T Therapy. Blood.

[116] Xu J, Chen LJ, Yang SS, Sun Y, Wu W, Liu YF (2019). Exploratory trial of a biepitopic CAR T-targeting B cell maturation antigen in relapsed/refractory multiple myeloma. Proc Natl Acad Sci U S A.

[117] Yan Z, Cao J, Cheng H, Qiao J, Zhang H, Wang Y (2019). A combination of humanised anti-CD19 and anti-BCMA CAR T cells in patients with relapsed or refractory multiple myeloma: a single-arm, phase 2 trial. Lancet Haematol.

[118] Li C, Mei H, Hu Y, Guo T, Liu L, Jiang H (2019). A Bispecific CAR-T Cell Therapy Targeting Bcma and CD38 for Relapsed/Refractory Multiple Myeloma: Updated Results from a Phase 1 Dose-Climbing Trial. Blood.

[119] Baumeister SH, Murad J, Werner L, Daley H, Trebeden-Negre H, Gicobi JK (2019). Phase I Trial of Autologous CAR T Cells Targeting NKG2D Ligands in Patients with AML/MDS and Multiple Myeloma. Cancer Immunol Res.

[120] Carrabba MG, Casucci M, Hudecek M, Quintarelli C, Briones J, Hajek R (2018). Phase I-IIa Clinical Trial to Assess Safety and Efficacy of MLM-CAR44.1, a CD44v6 Directed CAR-T in Relapsed/Refractory Acute Myeloid Leukemia (AML) and Multiple Myeloma (MM). Blood.

[121] Guo B, Chen M, Han Q, Hui F, Dai H, Zhang W (2016). CD138-directed adoptive immunotherapy of chimeric antigen receptor (CAR)-modified T cells for multiple myeloma. J Cell Immunother.

[122] Popat R, Zweegman S, Cavet J, Yong K, Lee L, Faulkner J (2019). Phase 1 First-in-Human Study of AUTO2, the First Chimeric Antigen Receptor (CAR) T Cell Targeting APRIL for Patients with Relapsed/Refractory Multiple Myeloma (RRMM). Blood.

[123] Ramos CA, Savoldo B, Torrano V, Ballard B, Zhang H, Dakhova O (2016). Clinical responses with T lymphocytes targeting malignancy-associated κ light chains. J Clin Invest.

[124] Smith EL, Harrington K, Staehr M, Masakayan R, Jones J, Long TJ, et al. GPRC5D is a target for the immunotherapy of multiple myeloma with rationally designed CAR T cells. Sci Transl Med. 2019;11(485). 10.1126/scitranslmed.aau7746 PMC7508042 30918115

[125] Stadtmauer EA, Faitg TH, Lowther DE, Badros AZ, Chagin K, Dengel K (2019). Long-term safety and activity of NY-ESO-1 SPEAR T cells after autologous stem cell transplant for myeloma. Blood Adv.

[126] Fernandez de Larrea C, Staehr M, Lopez A, Chen Y, Purdon TJ, Ng KY (2019). Optimal Dual-Targeted CAR Construct Simultaneously Targeting Bcma and GPRC5D Prevents Bcma-Escape Driven Relapse in Multiple Myeloma. Blood.

[127] Costello, C., N. Raje, N. Bahlis, B. Dholaria, M. Solh, M. Levy, et al. MAGNETISMM-1: Phase 1 study of elranatamab (PF-06863135), a B-Cell Maturation Antigen (BCMA) targeted CD3-engaging bispecific antibody for patienta with relapsed or refractory Multiple Myeloma (MM). (Oral communication). in European Hematology Association (EHA) Virtual Congress 2021 (June 09-17)*.* 2021.

[128] Krishnan, A.Y., J.G. Berdeja, A. Oriol, N.W.C.J. van de Donk, P. Rodríguez-Otero, E. Askari, et al. Talquetamab, a G Protein-coupled Receptor family C group 5 member D (GPRC5D) × CD3 bispecific antibody, in Relapsed/Refractory Multiple Myeloma: Updated results of a phase 1, first-in-human study. in European Hematology Association (EHA) Virtual Congress 2021 (June 09-17)*.* 2021.

[129] Cohen AD, Harrison SJ, Krishnan A, Fonseca R, Forsberg PA, Spencer A (2020). Initial Clinical Activity and Safety of BFCR4350A, a FcRH5/CD3 T-Cell-Engaging Bispecific Antibody, in Relapsed/Refractory Multiple Myeloma. Blood.

[130] Rajkumar SV, Kumar S (2020). Multiple myeloma current treatment algorithms. Blood Cancer J.

[131] Goldsmith SR, Fiala MA, Wang B, Schroeder MA, Wildes TM, Ghobadi A (2020). DCEP and bendamustine/prednisone as salvage therapy for quad- and penta-refractory multiple myeloma. Ann Hematol.

[132] Kazandjian D, Mo CC, Landgren O, Richardson PG (2020). The role of high-dose melphalan with autologous stem-cell transplant in multiple myeloma: is it time for a paradigm shift. Br J Haematol.

[133] Auner HW, Szydlo R, Rone A, Chaidos A, Giles C, Kanfer E (2013). Salvage autologous stem cell transplantation for multiple myeloma relapsing or progressing after up-front autologous transplantation. Leuk Lymphoma.

[134] Nieto Y, Valdez BC, Pingali SR, Bassett R, Delgado R, Nguyen J (2017). High-dose gemcitabine, busulfan, and melphalan for autologous stem-cell transplant in patients with relapsed or refractory myeloma: a phase 2 trial and matched-pair comparison with melphalan. Lancet Haematol.

[135] Dhakal B, D'Souza A, Kleman A, Chhabra S, Mohan M, Hari P. Salvage second transplantation in relapsed multiple myeloma. Leukemia. 2020. 10.1038/s41375-020-1005-8 PMC8020391 32747684

[136] Gertz MA, Lacy MQ, Inwards DJ, Gastineau DA, Tefferi A, Chen MG (2000). Delayed stem cell transplantation for the management of relapsed or refractory multiple myeloma. Bone Marrow Transplant.

[137] Gonsalves WI, Gertz MA, Lacy MQ, Dispenzieri A, Hayman SR, Buadi FK (2013). Second auto-SCT for treatment of relapsed multiple myeloma. Bone Marrow Transplant.

[138] Gössi U, Jeker B, Mansouri Taleghani B, Bacher U, Novak U, Betticher D (2018). Prolonged survival after second autologous transplantation and lenalidomide maintenance for salvage treatment of myeloma patients at first relapse after prior autograft. Hematol Oncol.

[139] Lemieux E, Hulin C, Caillot D, Tardy S, Dorvaux V, Michel J (2013). Autologous stem cell transplantation: an effective salvage therapy in multiple myeloma. Biol Blood Marrow Transplant.

[140] Manjappa S, Fiala MA, King J, Kohnen DA, Vij R (2018). The efficacy of salvage autologous stem cell transplant among patients with multiple myeloma who received maintenance therapy post initial transplant. Bone Marrow Transplant.

[141] Michaelis LC, Saad A, Zhong X, Le-Rademacher J, Freytes CO, Marks DI (2013). Salvage second hematopoietic cell transplantation in myeloma. Biol Blood Marrow Transplant.

[142] Sellner L, Heiss C, Benner A, Raab MS, Hillengass J, Hose D (2013). Autologous retransplantation for patients with recurrent multiple myeloma: a single-center experience with 200 patients. Cancer.

[143] Shah N, Ahmed F, Bashir Q, Qureshi S, Dinh Y, Rondon G (2012). Durable remission with salvage second autotransplants in patients with multiple myeloma. Cancer.

[144] Singh Abbi KK, Zheng J, Devlin SM, Giralt S, Landau H (2015). Second autologous stem cell transplant: an effective therapy for relapsed multiple myeloma. Biol Blood Marrow Transplant.

[145] Veltri LW, Milton DR, Delgado R, Shah N, Patel K, Nieto Y (2017). Outcome of autologous hematopoietic stem cell transplantation in refractory multiple myeloma. Cancer.

[146] Zannetti BA, Tacchetti P, Pantani L, Gamberi B, Tosi P, Rocchi S (2017). Novel agent-based salvage autologous stem cell transplantation for relapsed multiple myeloma. Ann Hematol.

[147] Goldschmidt H, Baertsch MA, Schlenzka J, Becker N, Habermehl C, Hielscher T, et al. Salvage autologous transplant and lenalidomide maintenance vs. lenalidomide/dexamethasone for relapsed multiple myeloma: the randomized GMMG phase III trial ReLApsE. Leukemia. 2020. 10.1038/s41375-020-0948-0 32694619

[148] Cook G, Royle KL, O'Connor S, Cairns DA, Ashcroft AJ, Williams CD (2019). The impact of cytogenetics on duration of response and overall survival in patients with relapsed multiple myeloma (long-term follow-up results from BSBMT/UKMF Myeloma X Relapse [Intensive]): a randomised, open-label, phase 3 trial. Br J Haematol.

[149] McCarthy PL, Holstein SA, Petrucci MT, Richardson PG, Hulin C, Tosi P (2017). Lenalidomide Maintenance After Autologous Stem-Cell Transplantation in Newly Diagnosed Multiple Myeloma: A Meta-Analysis. J Clin Oncol.

[150] Durie BGM, Hoering A, Abidi MH, Rajkumar SV, Epstein J, Kahanic SP (2017). Bortezomib with lenalidomide and dexamethasone versus lenalidomide and dexamethasone alone in patients with newly diagnosed myeloma without intent for immediate autologous stem-cell transplant (SWOG S0777): a randomised, open-label, phase 3 trial. Lancet.

[151] Bringhen S, D'Agostino M, Paris L, Ballanti S, Pescosta N, Spada S (2020). Lenalidomide-based induction and maintenance in elderly newly diagnosed multiple myeloma patients: updated results of the EMN01 randomized trial. Haematologica.

[152] Facon T, Dimopoulos MA, Dispenzieri A, Catalano JV, Belch A, Cavo M (2018). Final analysis of survival outcomes in the phase 3 FIRST trial of up-front treatment for multiple myeloma. Blood.

[153] Rajkumar SV (2016). *Updated Diagnostic Criteria and Staging System for Multiple Myeloma*. Am Soc Clin Oncol Educ Book.

[154] Moreau P, Zamagni E, Mateos MV (2019). Treatment of patients with multiple myeloma progressing on frontline-therapy with lenalidomide. Blood Cancer J.

[155] Mateos M-V, Berdeja JG, Dimopoulos MA, Siegel DS, Ho PJ, Huang M (2018). Efficacy and Safety of Carfilzomib and Dexamethasone in Lenalidomide Exposed and Refractory Multiple Myeloma Patients: Combined Analysis of Carfilzomib Trials. Blood.

[156] Siegel DS, Schiller GJ, Samaras C, Sebag M, Berdeja J, Ganguly S (2020). Pomalidomide, dexamethasone, and daratumumab in relapsed refractory multiple myeloma after lenalidomide treatment. Leukemia.

[157] Sonneveld P, Zweegman S, Cavo M, Nasserinejad K, Troia R, Pour L (2018). Carfilzomib, Pomalidomide and Dexamethasone (KPd) in Patients with Multiple Myeloma Refractory to Bortezomib and Lenalidomide. the EMN011 Trial. Blood.

[158] Dimopoulos MA, Weisel K, Moreau P, Anderson L, White DJ, San-Miguel JF (2018). Pomalidomide + Bortezomib + Low-Dose Dexamethasone Vs Bortezomib + Low-Dose Dexamethasone As Second-Line Treatment in Patients with Lenalidomide-Pretreated Multiple Myeloma: A Subgroup Analysis of the Phase 3 Optimismm Trial. Blood.

[159] Sanchez L, Barley K, Richter J, Franz J, Cho HJ, Jagannath S (2020). Immunomodulatory drug- and proteasome inhibitor-backbone regimens in the treatment of relapsed multiple myeloma: an evidence-based review. Expert Rev Hematol.

[160] Usmani SZ, Quach H, Mateos M-V, Landgren O, Leleu X, Siegel DS (2019). Carfilzomib, Dexamethasone, and Daratumumab Versus Carfilzomib and Dexamethasone for the Treatment of Patients with Relapsed or Refractory Multiple Myeloma (RRMM): Primary Analysis Results from the Randomized, Open-Label, Phase 3 Study Candor (NCT03158688). Blood.

[161] Orlowski RZ, Moreau P, Niesvizky R, Ludwig H, Oriol A, Chng WJ (2019). Carfilzomib-Dexamethasone Versus Bortezomib-Dexamethasone in Relapsed or Refractory Multiple Myeloma: Updated Overall Survival, Safety, and Subgroups. Clin Lymphoma Myeloma Leuk.

[162] Moreau P, Joshua D, Chng WJ, Palumbo A, Goldschmidt H, Hájek R (2017). Impact of prior treatment on patients with relapsed multiple myeloma treated with carfilzomib and dexamethasone vs bortezomib and dexamethasone in the phase 3 ENDEAVOR study. Leukemia.

[163] Bringhen S, Pour L, Vorobyev V, Vural F, Warzocha K, Benboubker L (2021). Isatuximab plus pomalidomide and dexamethasone in patients with relapsed/refractory multiple myeloma according to prior lines of treatment and refractory status: ICARIA-MM subgroup analysis. Leuk Res.

[164] Weisel K., Quach H., Nooka A., Samoylova O., Venner C. P., Kim K., et al., Carfilzomib, dexamethasone (kd) and daratumumab versus kd in relapsed or refractory multiple myeloma: subgroup analysis of the candor study by number of prior lines of therapy and prior therapies, in The 25th European Hematology Association Annual Congress (EHA25 Virtual). 2020.

[165] Gooding S, Lau IJ, Sheikh M, Roberts P, Wong J, Dickens E (2015). Double Relapsed and/or Refractory Multiple Myeloma: Clinical Outcomes and Real World Healthcare Costs. PLoS ONE.

[166] Usmani S, Ahmadi T, Ng Y, Lam A, Desai A, Potluri R (2016). Analysis of Real-World Data on Overall Survival in Multiple Myeloma Patients With ≥3 Prior Lines of Therapy Including a Proteasome Inhibitor (PI) and an Immunomodulatory Drug (IMiD), or Double Refractory to a PI and an IMiD. Oncologist.

[167] Lonial S, Weiss BM, Usmani SZ, Singhal S, Chari A, Bahlis NJ (2016). Daratumumab monotherapy in patients with treatment-refractory multiple myeloma (SIRIUS): an open-label, randomised, phase 2 trial. (1474-547X (Electronic)). Lancet.

[168] San Miguel JF, Weisel KC, Song KW, Delforge M, Karlin L, Goldschmidt H (2015). Impact of prior treatment and depth of response on survival in MM-003, a randomized phase 3 study comparing pomalidomide plus low-dose dexamethasone versus high-dose dexamethasone in relapsed/refractory multiple myeloma. Haematologica.

[169] Usmani S, Nahi H, Plesner T, Weiss B, Bahlis N, Belch A (2020). Daratumumab monotherapy in patients with heavily pretreated relapsed or refractory multiple myeloma: final results from the phase 2 GEN501 and SIRIUS trials. Lancet Haematol.

[170] Bringhen S, Attal M, Pour L, Vorobyev V, Vural F, Warzocha K (2019). *ICARIA-MM study: efficacy analysis according to prior lines of treatment*. Clin Lymphoma Myeloma Leuk.

[171] Chari A, Martinez-Lopez J, Mateos MV, Bladé J, Benboubker L, Oriol A (2019). Daratumumab plus carfilzomib and dexamethasone in patients with relapsed or refractory multiple myeloma. Blood.

[172] Bringhen S, Mina R, Cafro AM, Liberati AM, Spada S, Belotti A (2018). Once-weekly carfilzomib, pomalidomide, and low-dose dexamethasone for relapsed/refractory myeloma: a phase I/II study. Leukemia.

[173] Yang Y, Li Y, Gu H, Dong M, Cai Z (2020). Emerging agents and regimens for multiple myeloma. J Hematol Oncol.

[174] Cedena MT, Martin-Clavero E, Wong S, Shah N, Bahri N, Alonso R (2020). The clinical significance of stringent complete response in multiple myeloma is surpassed by minimal residual disease measurements. PLoS ONE.

[175] Gagelmann N, Riecken K, Wolschke C, Berger C, Ayuk FA, Fehse B (2020). Development of CAR-T cell therapies for multiple myeloma. Leukemia.

[176] Chari A, Richardson PG, Romanus D, Dimopoulos MA, Sonneveld P, Terpos E (2020). Real-world outcomes and factors impacting treatment choice in relapsed and/or refractory multiple myeloma (RRMM): a comparison of VRd, KRd, and IRd. Expert Rev Hematol.

[177] Chari A, Romanus D, Palumbo A, Blazer M, Farrelly E, Raju A (2020). Randomized Clinical Trial Representativeness and Outcomes in Real-World Patients: Comparison of 6 Hallmark Randomized Clinical Trials of Relapsed/Refractory Multiple Myeloma. Clin Lymphoma Myeloma Leuk.

[178] Bruno AS, Willson JL, Opalinska JM, Nelson JJ, Lunacsek OE, Stafkey-Mailey DR (2020). Recent real-world treatment patterns and outcomes in US patients with relapsed/refractory multiple myeloma. Expert Rev Hematol.

[179] Richardson PG, San Miguel JF, Moreau P, Hajek R, Dimopoulos MA, Laubach JP (2018). Interpreting clinical trial data in multiple myeloma: translating findings to the real-world setting. Blood Cancer J.

[180] Terpos E, Engelhardt M, Cook G, Gay F, Mateos MV, Ntanasis-Stathopoulos I (2020). Management of patients with multiple myeloma in the era of COVID-19 pandemic: a consensus paper from the European Myeloma Network (EMN). Leukemia.

[181] Pawlyn C, Davies FE (2019). Toward personalized treatment in multiple myeloma based on molecular characteristics. Blood.

